# The Internal Relation between Quantum Chemical Descriptors and Empirical Constants of Polychlorinated Compounds

**DOI:** 10.3390/molecules23112935

**Published:** 2018-11-10

**Authors:** Jiangchi Fei, Qiming Mao, Lu Peng, Tiantian Ye, Yuan Yang, Shuang Luo

**Affiliations:** 1 Institute of Environmental Engineering, School of Metallurgy and Environment, Central South University, Changsha 410083, China; jack-fei@csu.edu.cn; 2 College of Resources and Environment, Hunan Agricultural University, Changsha 410128, China; qimingmao@126.com; 3 College of Biology and Environmental Sciences, Jishou University, Jishou 416000, China; abonluo@gmail.com; 4 South China Institute of Environmental Sciences, Ministry of Environmental Protection, Guangzhou 510655, China; yetiantian@scies.org

**Keywords:** quantum chemical descriptor, Hammett constant, meta-position, prediction model

## Abstract

Quantum chemical descriptors and empirical parameters are two different types of chemical parameters that play the fundamental roles in chemical reactivity and model development. However, previous studies have lacked detail regarding the relationship between quantum chemical descriptors and empirical constants. We selected polychlorinated biphenyls (PCBs) as an object to investigate the intrinsic correlation between 16 quantum chemical descriptors and Hammett constants. The results exhibited extremely high linearity for $\sum \sigma_{o,\;m,\;p}^{+}$ with Q_xx/yy/zz_, *α* and *E*_HOMO_ based on the meta-position grouping. Polychlorinated dibenzodioxins (PCDDs) and polychlorinated naphthalenes (PCNs) congeners, as two independent compounds, validated the reliability of the relationship. The meta-substituent grouping method between $\sum \sigma_{o,\;m,\;p}^{+}$ and *α* was successfully used to predict the rate constant (*k*) for ^•^OH oxidation of PCBs, as well as the octanol/water partition coefficient (log*K*_OW_) and aqueous solubility (−log*S*_W_) of PCDDs, and exhibited excellent agreement with experimental measurements. Revealing the intrinsic correlation underlying the empirical constant and quantum chemical descriptors can develop simpler and higher efficient model application in predicting the environmental behavior and chemical properties of compounds.

## 1. Introduction

Computational chemistry is defined as a mathematical description of chemistry that is an effective tool to investigate the kinetics and rate constant of chemical reactions, develop a predictive model, and calculate the properties of molecules to obtain some quantum chemical descriptors [1]. Quantum chemical descriptors play a fundamental role in chemistry, environmental protection, pharmaceutical science, and health research [2], as they identify the correlations between chemical structures and properties (i.e., quantitative structure−activity relationship, QSAR) [3,4,5,6]. A large number of geometrical, electrostatic, and quantum information regarding molecules can be presented by computational chemistry software. Thus, many descriptors reflect the properties of molecules and can provide insight into the chemical nature of compounds under given reaction conditions [7,8,9]. For example, the descriptors *E*_LUMO_ (energy of lowest unoccupied molecular orbital) and *E*_HOMO_ (highest occupied molecular orbital) reflect the molecular orbital energies [1,10,11], which play an important role in predominating many chemical reactions and determining molecular electronic transition [1,12]. The *E*_HOMO_ and *E*_LUMO_ are directly related to the ionization potential and electron affinity, and characterize the susceptibility of molecules toward attack by electrophiles and nucleophiles, respectively [1]. Another descriptor polarizability is dependent on the electron distribution of the entire molecule, which determines the dynamical response to external fields, and provides insight into a molecular internal structure [13,14].

Molecular descriptors derived from quantum chemical calculations have been widely used for the prediction and interpretation of quantitative aspects of organic reactions [1,15,16,17,18]. For example, Luo et al. [19] investigated the UV direct photolysis of ibuprofen and sulfamethoxazole based on experimental measurements, and further accounted for its mechanism based on *E*_LUMO_-*E*_HOMO_ descriptor. The small *E*_LUMO_-*E*_HOMO_ gap values presented the lower excitation energy and higher quantum yield, which accounted for the high photolysis rate value [19]. Xiao et al. developed a QSAR model to predict the second-order rate constants for ${\mathrm{SO}}_{4}^{•-}$ degradation of emerging micro-pollutants ($k_{{\mathrm{SO}}_{4}^{•-}}$) based on the ratio of oxygen to carbon atoms (#O:C) and the *E*_LUMO_-*E*_HOMO_: ln$k_{{\mathrm{SO}}_{4}^{•-}}$ = 26.8 − 3.97 × (#O:C) − 0.75 × (*E*_LUMO_-*E*_HOMO_). The model provided a robust predictive tool for estimating emerging micropollutants removal by SO_4_^•−^ mediated process [20]. More importantly, the QSAR model combining quantum chemical descriptors and experimental data can predict unobserved chemical phenomena in some cases. Although quantum chemical descriptors can provide a more accurate and detailed description of electronic effects than empirical methods, quantum chemical descriptors calculated at a higher level of theory are still difficult to obtain [21,22]. Thereby, chemical descriptor calculation is an expensive and difficult process, which limits the high-efficiency prediction at the screening level [21,22,23]. Furthermore, another type of empirical parameter is determined by experimentations under the same experimental constraints and controls, and a common understanding of measurement. An empirical parameter is a similar effect on the properties or reactivity of each compound in a series of structurally related compounds [24], such as acid dissociation constant (*p*Ka), octanol/water partition coefficient (log*k*_OW_), and substituent constants. As an important empirical constant, the Hammett substituent constants (*σ*) has provided insight into the relationship between reactivity and chemical structures containing aromatic rings [25]. Although this type of constant is reckoned to be accurate, simple, and have a low computational cost, it still neglects the isomers and the steric effects that exert a great influence on chemical activity [23,26]. Instead, the QSAR model reflects the structural and chemical reactivity of the molecule and exhibits advantages for an empirical constant model [14,20,27]. For instance, Russell et al. [28] revealed that Henry’s law constant could be approximated as a linear function of factors related to bulk, lipophilicity, and polarity based on 63 molecular structures. Overall, both the QSAR model and empirical constants reflect the relationship regarding structure-activity of compounds [29]; there may be a connection between the quantum chemical descriptor and empirical constant. The intrinsic relationship underlying the quantum chemical descriptor and empirical constant still needs to be revealed.

Thus, how to combine the advantage of quantum chemical descriptors and empirical constants to develop an efficient, accurate, and simple model is quite meaningful to study. However, there rarely have been other reports about the relationship between quantum chemical descriptors and empirical constants are rare. Santiago et al. [25] developed a mathematical modeling approach to incorporate steric effects in Hammett-type correlations. They found a strong correlation between the Hammett values of para-substitution and natural bond orbital (NBO) charges (*R*^2^ = 0.96). The Hammett values can be used as an alternative to NBO charges [25]. Our previous study had tried to trap the relationship among polychlorinated compounds between polarizability (*α*, a quantum chemical descriptor) and Hammett constant (*σ*, an empirical constant) [21], which based on two good models (log*k* = −11.6 − 1.39 × $\sum \sigma_{o,\;m,\;p}^{+}$ [30]) and *α* (ln*k* = −0.054 × *α* − 19.49 [14]) to predicted the kinetics of ^•^OH oxidation of PCBs (*k* values) in gas-phase. However, the findings were haphazard and limited.

Revealing the relationships hidden in quantum chemical descriptors and empirical constants will greatly improve the efficiency and accuracy of the prediction model. More importantly, the revelation relationship will disclose the intrinsic correlation between structure and apparent experiment. In this study, we selected a class of polychlorinated compounds, polychlorinated biphenyls (PCBs) as an object because of 210 compounds with similar structures and multiple substitution positions, in order to investigate the relationships between 16 quantum chemical descriptors and Hammett constants. Another two classes of polychlorinated compounds, polychlorinated dibenzodioxins (PCDDs) and polychlorinated naphthalenes (PCNs) congeners, were selected to validate the obtained relationship. To reveal the intrinsic correlation underlying empirical constants and quantum chemical descriptors can provide a simpler and higher efficient method with great application potential for model development. The result will help develop fast and tractable prediction power in predicting the phenomenon of polychlorinated compounds involving environmental pollution and chemical properties.

## 2. Results and Discussion

### 2.1. Reveal Relationships between Quantum Descriptors and Hammett Constants

The Hammett constant (including *σ*, *σ^+^* and *σ^−^*) is a reflection of the electronic nature and position of the substituent [31]. There have been multiple positions substituted by Cl atoms at the ortho-, meta-, and para-positions, respectively (Figure S1). Thus, the $\sum \sigma_{o,\;m,\;p}^{\;}$, $\sum \sigma_{o,\;m,\;p}^{+}$, and $\sum \sigma_{o,\;m,\;p}^{-}$ values are the sum of all of the substituent constants of the Cl atoms attached to the aromatic ring, respectively corresponding to $\sigma_{o,\;m,\;p\;}^{\;}$, $\sigma_{o,\;m,\;p\;}^{+}$ and $\sigma_{o,\;m,\;p}^{-}$ values (the o, m, and p represent the substitution on the ortho-, meta- and para-positions, respectively) (in Table S1) [6,31]. Sixteen quantum chemical descriptors (Table S2) were obtained from the optimized results.

**Figure 1:**
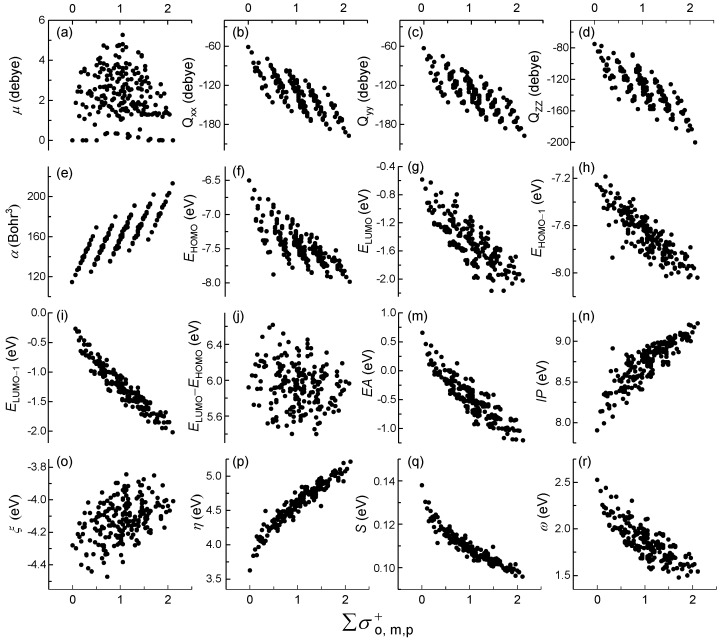
Relationships of 16 quantum chemical descriptors (**a**–**r**) and ∑$\sigma_{o,\;m,\;p}^{+}$ for polychlorinated biphenyls (PCBs) congeners. The (**a**), (**b**) referred to our previous study [21].

For 210 PCBs congeners, the relationships among 16 quantum chemical descriptors with $\sum \sigma_{o,\;m,\;p}^{\;}$, $\sum \sigma_{o,\;m,\;p}^{+}$, and $\sum \sigma_{o,\;m,\;p}^{-}$ values were listed in Figure S2, Figure 1 and Figure S3, respectively. The different values of ∑$\sigma_{o,\;m,\;p}$, ∑$\sigma_{o,\;m,\;p}^{+}$, and ∑$\sigma_{o,\;m,\;p}^{-}$ values were 72, 74 and 44, respectively. In other words, many different structures of PCBs congeners were faced with the same sigma values. The results showed that there have been significantly different trends among ∑$\sigma_{o,\;m,\;p}$, ∑$\sigma_{o,\;m,\;p}^{+}$, and ∑$\sigma_{o,\;m,\;p}^{-}$ for the same quantum chemical descriptors. There are five obvious five groups of linear correlation pattern of ∑$\sigma_{o,\;m,\;p}^{+}$ and *Q*_xx__/yy/zz_, *α*, and *E*_HOMO_, respectively. However, the single linear trend for ∑$\sigma_{o,\;m,\;p}$ and the vertical trend were shown for ∑$\sigma_{o,\;m,\;p}^{-}$, respectively. All of relationship for others descriptors with ∑$\sigma_{o,\;m,\;p}$, ∑$\sigma_{o,\;m,\;p}^{+}$ and ∑$\sigma_{o,\;m,\;p}^{-}$ values were scatter distribution, except for *η* and S converging toward a baseline. It’s worth noting that only a single linear for 210 congers did not distinguish more information relative to the five obvious linear correlation groups. It is worth mentioning that $\sigma_{o}^{-}=\sigma_{p}^{-}$ and $\sigma_{m\;}^{-}{=\;2\sigma }_{p}^{-}={2\sigma }_{o}^{-}$, which caused the points of ∑$\sigma_{o,\;m,\;p}^{-}$ and descriptors concentrated distribution into 15 approximations and hid more discrepant information. Thus, the high relationships among *Q*_xx__/yy/zz_, *α*, *E*_HOMO_ and ∑$\sigma_{o,\;m,\;p}^{+}$ were selected for further analysis in this study, respectively.

### 2.2. Mechanistic Interpretation of Internal Relationship

**Figure 2:**
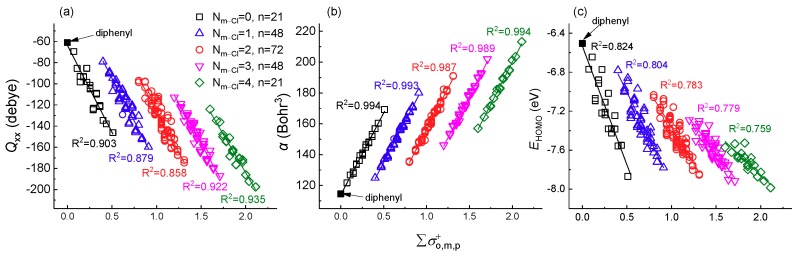
The relationship of $\sum \sigma_{o,\;m,\;p}^{+}$ and quadrupole moment tensor along the x axis (*Q*_xx_), polarizability (*α*) [21] and energy of the highest occupied molecular orbital (*E*_HOMO_) for PCBs congeners. The N_m-Cl_ represents the number of Cl atoms substituted at the meta-position, ranging from 0 to 4 with the number of congeners n = 21, 48, 72, 48, and 21, respectively. (**a**)$\;\sum \sigma_{o,\;m,\;p}^{+}$ and *Q*_xx_; (**b**)$\;\sum \sigma_{o,\;m,\;p}^{+}$ and *α*; (**c**) $\sum \sigma_{o,\;m,\;p}^{+}$ and *E*_HOMO_.

The further investigation result showed that *Q*_xx__/yy/__zz_, *α*, and *E*_HOMO_ all displayed extremely high linearly correlation to $\sum \sigma_{o,\;m,\;p}^{+}$ (Figure 2 and Figure S4). The result showed that all of the PCBs congeners are classified into five clusters according to the number of Cl atoms substituted at the meta-position (N_m__-Cl_) on the ring. As shown in Figure 2, there are 21, 48, 72, 48, and 21 congeners in each group for meta-position with N_m__-Cl_ ranging from 0 to 4, respectively. In each meta-position cluster, *Q*_xx__/yy/__zz_ and *E*_HOMO_ values decrease with the increase of $\sum \sigma_{o,\;m,\;p}^{+}$ while *α* values increase with the increase of $\sum \sigma_{o,\;m,\;p}^{+}$. The *R*^2^ for *Q*_xx__/yy/__zz_, and *α* with $\sum \sigma_{o,\;m,\;p}^{+}$ were 0.836~0.935 and 0.987~0.994, respectively, which were higher the *R*^2^ for *E*_HOMO_ with $\sum \sigma_{o,\;m,\;p}^{+}$ (0.759~0.824). The extremely high *R*^2^ (>0.7) indicated that the meta-position chlorination on PCBs congeners play a crucial role in the relationship between $\sum \sigma_{o,\;m,\;p}^{+}$ and *Q*_xx/yy/zz_, and *α* and *E*_HOMO_. Furthermore, the trend for *Q*_xx/yy/zz_, *α*, and *E*_HOMO_ values also supported that meta-position determined the reactivity of PCBs. The highly linear correlation illustrated that the simple $\sum \sigma_{o,\;m,\;p}^{+}$ can be used to explain or substitute complex quantum chemical descriptors, such as *Q*_xx/yy/zz_, *α*, and *E*_HOMO_, based on meta-substitute grouping. Their corresponding fitted linear equations were listed in Table 1 ($\sum \sigma_{o,\;m,\;p}^{+}$ and *Q*_xx_, *α*, *E*_HOMO_) and Table S3 ($\sum \sigma_{o,\;m,\;p}^{+}$ and *Q*_yy_, *Q*_zz_).

In order to gain insights into their connections, the intrinsic characters of *Q*_xx/yy/zz_, *α* and *E*_HOMO_ need further to be further revealed. The $\sum \sigma_{o,\;m,\;p}^{+}$ is an empirical value reflecting the electronic nature and position of the substituent [31]. The quadrupole moment (*Q*_xx_, *Q*_yy_, *Q*_zz_) reflects the distribution of the molecular charge in the x-, y-, and z-coordinates or the departure degree relative to the spherical-symmetry [32]. The polarizability (*α*) is defined as the ratio of the induced dipole moment of a molecule to the electric field that produces its dipole moment [33], which is an important electronic descriptor to reflect the electron distribution in the molecule [34] that is well correlated to the overall reactivity of molecule [13,35,36]. *E*_HOMO_ characterizes the susceptibility of a molecule toward attack by electrophiles. A molecule with higher *E*_HOMO_ is more reactive to attack by strong electrophiles [14,21]. Some investigations have shown that *Q*_xx/yy/zz_, *α* and *E*_HOMO_ are associated with many chemical activities and properties. For instance, Kim and Mhin et al. suggested that the change in the polarity of the quadrupole moment (*Q*_xx/yy/zz_) was related to the reduction of the repulsive interaction, which played a vital role in governing the geometry of aromatics [37,38]. The investigation reported by Zeng et al. indicated that the quadrupole moment (*Q*_yy_ and *Q*_zz_) were successfully used to develop a model for predicting the *n*-octanol/water partition coefficients (log*K*_OW_) and aqueous solubility coefficients(−log*S*_W_) of PCDDs [39,40]. In addition, our previous study developed a QSAR model to predict the ^•^OH degradation of PCBs based on single descriptor *α*. The *α* played an important role in determining the reaction rate (*k*) [14]. Luo et al. suggested that the more polarizable (*α*) the molecule, the easier an approaching electrophile (or nucleophile) can distort the electron density of the aromatic molecule increasing the rate of reaction [21]. For *E*_HOMO_ descriptor, Yan et al. developed a QSAR model for ^•^OH oxidation of the multiring hydrocarbon in the gas‒phase based on partial least squares regression [41]. They reported that *E*_HOMO_ was the most suitable for model development and the higher *E*_HOMO_ value corresponds to higher reactivity. Thus, ∑$\sigma_{o,\;m,\;p}^{+}$ can be considered as the intuitive experimental phenomenon of the structure descriptor, as Q_xx/yy/zz_, *α* and *E*_HOMO,_ and so on.

**Figure 3:**
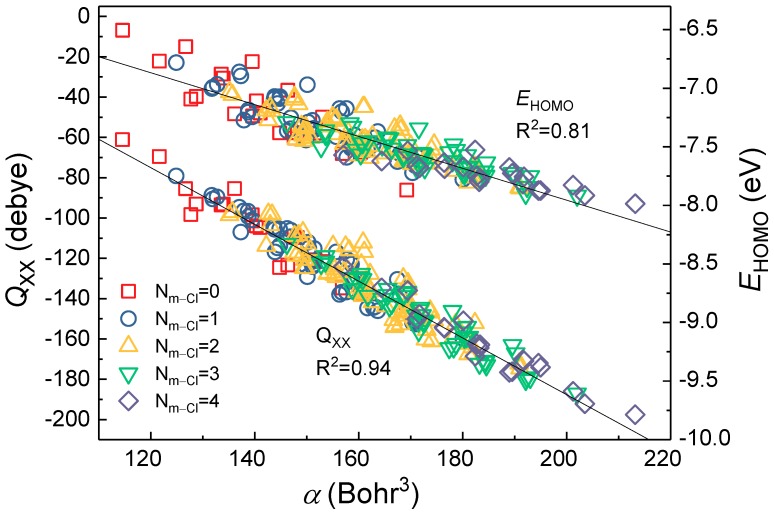
The relationship of *α* with Q_xx_ and *E*_HOMO_ of PCBs congeners.

Due to Q_xx/yy/zz_, *α*, and *E*_HOMO_ having highly correlated to ∑$\sigma_{o,\;m,\;p}^{+}$, there may be collinearity between *Q*_xx/yy/zz_, *α*, and *E*_HOMO_. Further, Figure 3 showed that *α* with *Q*_xx_ and *E*_HOMO_ had a high correlation, with corresponding *R*^2^ values of 0.94 and 0.81, respectively. Meanwhile, *α* and *Q*_yy_/*Q*_zz_ had similarly high correlation (in Figure S5, the *R*^2^ values were 0.92 and 0.95, respectively). The quadrupole moment (*Q*_xx_, *Q*_yy_, *Q*_zz_) and *α* reflect the electron behavior and the homogeneity in the electronic properties of the molecule. The *α*, as one of the molecular electrostatic descriptors [14,20], is the principal factor determining the structure-activity relationship, even though *E*_HOMO_ represents the electron-donating power of the molecule [42]. Yang et al. suggested that the *E*_HOMO_ reflected only a single aspect of the molecule, while the *α* incorporated a number of molecular features [14].

The reasons for good performance with aromatic meta-substituent grouping regarding the relationships of quantum chemical descriptors and $\sum \sigma_{o,\;m,\;p}^{+}$ need to be further discussed. First of all, the $\sigma_{\;m}^{+}$ value (0.4) over $\sigma_{\;p}^{+}$ (0.11) and $\sigma_{\;o}^{+}$ (0.073) for Cl substituents was probably attributed to the meta-position, which determined its dominant role and showed the high correlation [21]. Another important reason, since Cl atoms are substituted on aromatic rings, is electron withdrawing through the *σ*-bond, which decreases the ring electron density in the Cl atoms’ substituted site [9,14]. The Cl atoms that were substituted at the meta-position can pull electrons from the aromatic ring, resulting in decreased electron density and suppressed HOMO distribution [14,21]. The HOMO distribution is the most direct reflection of the changes in electron distribution. Figure S6 listed the HOMO distribution of Cl atoms with different N_m-Cl_ numbers at meta-position and N_o-Cl_ at ortho-position. Luo et al. investigated the changed of HOMO distribution and Cl atoms at the meta-position [21]. The HOMO distribution of Cl atoms at the meta-position increased independent of the increasing number of Cl atoms at the meta-position. However, with the increasing number of Cl atoms at the meta-position, the HOMO distribution of the 1-, 2-, 6-, 1′-, 2′-, 6′-positions in the biphenyl ring (PCB15, PCB28, PCB100, and PCB155) was easily distorted and greatly varied. For the Cl atoms increasing at the meta-position and ortho-position, the Cl atoms at the meta-position (PCB15, PCB37, PCB81, PCB126, and PCB169) do not change the HOMO distribution in the biphenyl junction. However, once Cl atoms are added at the ortho-position (PCB66, PCB123, PCB167, and PCB189), their HOMO distribution of biphenyl junction and meta-position were greatly influenced and obviously changed. It is helpful to deepen our understanding of why meta-position played an important role in correlating *α* to $\sum \sigma_{o,\;m,\;p}^{+}$ in high linearity. However, the HOMO distribution is easily distorted and greatly varied when Cl atoms are substituted in other positions [21].

### 2.3. Application of Meta-Substituent Grouping

#### 2.3.1. Application in Similar Compounds

The aromatic meta-substituent grouping method was suitable for the application of PCBs congeners; however, a good method should also be applied to other similar compounds. Thus, in order to confirm the aromatic meta-substituent grouping method, we examined the relationships of $\sum \sigma_{o,\;m,\;p}^{+}$ and quantum chemical descriptors (Q_xx/yy/zz_, *α* and *E*_HOMO_) with Cl atoms substituted at meta-position for PCDDs and PCNs congeners (Figures S7 and S8). For PCDDs and PCNs with only 25 different $\sum \sigma_{o,\;m,\;p}^{+}$ values, the trends in the relationships of $\sum \sigma_{o,\;m,\;p}^{+}$ and Q_xx/yy/zz_, *α*, and *E*_HOMO_ were similar to those of the PCBs based on aromatic meta-substituent grouping. For PCDD, these parallel lines exhibited extremely high linearity for $\sum \sigma_{o,\;m,\;p}^{+}$ with *α* (*R*^2^ = 0.994~0.999) and *E*_HOMO_ (*R*^2^ = 0.982~0.999); however, the linearity for $\sum \sigma_{o,\;m,\;p}^{+}$ and Q_xx/yy/zz_ were acceptable (*R*^2^ = 0.712~0.999) (Table S8). For PCNs, the parallel lines Q_xx/yy/zz_ and *α* also exhibited extremely high linearity (*R*^2^ = 0.883~0.999) except for *E*_HOMO_, while the linearity of *E*_HOMO_ was not obvious (*R*^2^ = 0.438~0.677) (Table S9). The reason may be that the HOMO distribution is severely disturbed by the naphthalene ring structure relative to the biphenyl structure for PCBs and the dibenzodioxin structure for PCDDs, especially for the alpha positions in the naphthalene ring. The overall trends of Q_xx/yy/zz_, *α* and *E*_HOMO_ with $\sum \sigma_{o,\;m,\;p}^{+}$ are correlated to the number of Cl atoms substituted on the meta-position as well. The validation results of PCNs and PCDDs support the application domain in aromatic compounds based on the meta-substituent grouping method.

#### 2.3.2. Application in Prediction Model

Our previous study was the first to predict the *k* values of ^•^OH degradation of PCBs in the gas-phase based on the QSAR model and *α* [21]. The observed ln*k* values of ^•^OH oxidation of PCBs congeners (as validation data) were listed in Table S6. The result showed that the prediction *k* values were excellently consistent with experimental measurements (the validation coefficient *Q*^2^ = 0.825, the standard deviation Δlnk = −0.430~0.626, and the average of standard deviation $\bar{\Delta \mathrm{lnk}}$ = −0.03), and exhibited greater predictive power and convenience than the QSAR model for single *α* descriptor (Table 2). We also developed the meta-substituent grouping model to predict the log*K*_OW_ and −log*S*_W_ of PCDDs based on the existent quantum chemical descriptor model (log*K*_OW_ = 0.03345 × *α* + 0.39092 and −log*S*_W_ = 0.0693 × *α* − 3.6425) (Table 2) and observed values (Table S7) in this study [43,44,45]. The results showed that the standard deviation Δlog*K*_OW_ and Δ−log*S*_W_ ranged from −0.15 to 0.92 and from −0.25 to −1.45, and the average of standard deviation $\bar{{\Delta \log K}_{\mathrm{OW}}}$ and $\bar{\Delta -{\log S}_{W}}$ were 0.45 and −0.92, respectively. The *Q*^2^ between the prediction and observation values were 0.954 and 0.981, respectively. All of the models showed that the *p* < 0.01. These statistical diagnostics demonstrated that the predicted values of log*K*_OW_ and −log*S*_W_ were very accurate, which indicated that the method of combining the empirical Hammett constant and quantum-chemical descriptor based on meta-substituent grouping showed fast and tractable prediction power and a great application potential for model development.

## 3. Methods

### 3.1. Data Collection

The experimental *k* values of ^•^OH oxidation of PCBs (294–300 K) in gas-phase were obtained from previous study [21]. The experimental log*K*_OW_ and −log*S*_W_ values were collected from the studies of Huang [44] and Kim [45] et al. studies. For PCBs, PCDDs, and PCNs, these comprise in total 210, 76, and 76 congeners from non to fully (decachloro) Cl substituted on the benzene or naphthalene ring, respectively. Although diphenyl, dibenzo-1,4-dioxin and naphthalene did not have the substituted Cl atoms, they were still investigated in this study due to the structural similarities to PCBs, PCDDs and PCNs, respectively. The Cl substituted PCBs are classified by para (4-, 4′- position), meta (3-, 5-, 3′-, 5′- position), and ortho (2-, 6-, 2′-, 6′- position) substitution patterns, and the PCDDs and PCNs are classified by meta (2-, 3-, 7-, 8- position), and ortho (1-, 4-, 6-, 9- position) substitution patterns (without para-position) (Figure S1). The Hammett constant $\sum \sigma_{o,\;m,\;p}^{\;}$, $\sum \sigma_{o,\;m,\;p}^{+}$ and $\sum \sigma_{o,\;m,\;p}^{-}$ values are the sum of the substituent constants $\sigma_{o}^{\;}$ + $\sigma_{m}^{\;}$ + $\sigma_{p}^{\;}$, $\sigma_{o}^{+}$ + $\sigma_{m}^{+}$ + $\sigma_{p}^{+}$, and $\sigma_{o}^{-}$ + $\sigma_{m}^{-}$ + $\sigma_{p}^{-}$ respectively, which are the Cl atoms substituted to the aromatic ring on the ortho−, meta−, and para−positions, respectively [6,31]. The *σ*^+^ and *σ*^−^ constants represent the compounds with delocalized positive and negative charges, respectively based upon the heterolysis reaction of para-substituted cumyl chlorides and phenols [6,46]. The Hammett constants (*σ*, *σ^−^*, *σ^+^*) were described in the Supplementary Materials (Text S1) and their values are listed in Table S1 in detail.

### 3.2. Quantum Chemical Descriptors Calculation

The structures of 210 PCB congeners, 76 PCDDs, and 76 PCNs congeners (Tables S3–S5) were created by GaussView 5.0 [47]. First, the global minimum energy was optimized at Spartan’10 program [48] using the MMFF (Merck Molecular Force Field) method [49,50]. Then, the geometries were performed to further optimize in the gas‒phase using Gaussian 09 (Revision C.01) [51] at the mPW1PW91 (modified Perdew–Wang exchange and Perdew–Wang 91) hybrid density functional [52,53] combination with the MIDIX+ basis set [54,55]. It is reported that the MIDIX+ basis set had a good performance-to-cost ratio for the geometrical, orbital energy and electrostatic calculations in aromatic compounds [14,21,56]. All of the optimization structures were the local minima on potential energy surfaces with positive vibration frequencies. Sixteen quantum chemical descriptors, including the molecular dipole moment (*μ*), energy of the highest occupied molecular orbital (*E*_HOMO_), and energy of the lowest unoccupied molecular orbital (*E*_LU__MO_), energy of the second HOMO and LUMO (*E*_HOMO−1_ and *E*_LUMO+1_), gap of *E*_LUMO_ and *E*_HOMO_ (*E*_LUMO_−*E*_HOMO_), polarizability (*α*), electron affinity (*EA*), ionization potential (*IP*), quadrupole moment tensor along the x/y/z axis (*Q*_xx_*/Q*_yy_*/Q*_zz_), softness (*S*), electronegativity (*ζ*), hardness (*η*), and electrophilicity index (*ω*) were obtained from the optimized results. The descriptors and their formulas were introduced in detail in Table S2.

### 3.3. Model Development

The multilinear regression (MLR) analysis [57] was used to develop the meta-substituent grouping models in this study. We selected the compounds with experimental measurements (26 ln*k*, 17 log*K*_OW_ and 15 log*S*_W_ values) to validate the predictive power based on the meta-substituent relationship (Tables S6 and S7). The standard deviation and average of the standard deviation of prediction values represent the error between the experimental and predicted values. The determination coefficient *R*^2^ measures the observation value repeatability of the model, and the validation coefficient *Q*^2^ reflects the correlation between predicted values and observed values. The *Q*^2^ was calculated as following:

(1) $$\;Q^{2}\;=\;\frac{\sum_{i=1}^{n}{\left({\hat{y}}_{i}-{\bar{y}}_{i}\right)}^{2}}{\sum_{i=1}^{n}{\left(y_{i}-{\bar{y}}_{i}\right)}^{2}}\;$$

where *y_i_* and ${\hat{y}}_{i}$ are the observed and predicted values, respectively, and $\bar{y}$ was the average values of the predicted values. High *R*^2^ and *Q*^2^ values indicate a model with robust performance and good predictive power, respectively. In addition, the *R*^2^ and *Q*^2^ > 0.7 indicates the method with a better predictive performance. The statistical analyses were conducted using SPSS software version 17.0 [58]. 

## 4. Conclusions

In this study, we selected the PCBs as an object to investigate the relationships of 16 quantum chemical descriptors and Hammett constants in order to reveal their intrinsic correlation. By systematically analyzing the relationship of 16 quantum chemical descriptors and the Hammett relationship ($\sum \sigma_{o,\;m,\;p}^{+}$, $\sum \sigma_{o,\;m,\;p}^{\;}$ and $\sum \sigma_{o,\;m,\;p}^{-}$) for PCBs congeners, a very good correlation of $\sum \sigma_{o,\;m,\;p}^{+}$ with Q_xx/yy/zz_, *α*, and *E*_HOMO_ based on meta-position grouping were observed. PCDDs and PCNs as two independent compounds validated the reliability of the relationship in aromatic compounds based on the meta-substituent grouping. Furthermore, the meta-substituent grouping method between $\sum \sigma_{o,\;m,\;p}^{+}$ and quantum chemical descriptors was successfully used for apply in predicting ln*k* values for ^•^OH oxidation of PCBs, as well as the log*K*_OW_ and −log*S*_W_ of PCDDs, which exhibit excellent agreement with experimental measurements. The results indicated that combining empirical constants and quantum chemical descriptors based on meta-substituent grouping has greater tool application for predicting the environmental behavior and chemical properties of compounds.

## Figures and Tables

**Table 1:** The fitted linear equations of $\sum \sigma_{o,\;m,\;p}^{+}$ with *Q*_xx_, *α* and *E*_HOMO_ for PCBs congeners.

#	$\sum \sigma_{o,\;m,\;p}^{+}\;\mathrm{Range}$	$Q_{\mathrm{xx}}\;=\;A\;\times \;\sum \sigma_{o,\;m,\;p}^{+}+\;B$			$\alpha \;=\;A\;\times \;\sum \sigma_{o,\;m,\;p}^{+}+\;B\;$ [21]			$E_{\mathrm{HOMO}}\;=\;A\;\times \;\sum \sigma_{o,\;m,\;p}^{+}+\;B$		
		A	B	*R* ^2^	A	B	*R* ^2^	A	B	*R* ^2^
N_m-Cl_ = 0	0~0.51	−164.46	−63.53	0.903	106.13	113.94	0.994	−2.44	−6.53	0.824
N_m-Cl_ = 1	0.4~0.91	−162.23	−13.51	0.879	106.38	81.72	0.993	−1.88	−6.06	0.804
N_m-Cl_ = 2	0.8~1.31	−156.71	31.14	0.858	107.73	48.49	0.987	−1.49	−5.88	0.783
N_m-Cl_ = 3	1.2~1.71	−152.50	72.28	0.922	107.67	16.33	0.989	−1.20	−5.84	0.779
N_m-Cl_ = 4	1.6~2.11	−144.53	104.95	0.935	108.87	−17.84	0.994	−0.87	−6.12	0.759

**Table 2:** The model based on $\sum \sigma_{o,\;m,\;p}^{+}$ and meta-substituent grouping for predicting ^•^OH oxidation of PCBs, log*K*_OW_ and −log*S*_W_ of polychlorinated dibenzodioxins (PCDDs).

Congeners		Model	Statistical Diagnostic
PCBs	N_m-Cl_ = 0	ln*k* = ‒5.73 × $\sum \sigma_{o,\;m,\;p}^{+}$ − 25.64	$\bar{\Delta \mathrm{lnk}}$ = −0.03
	N_m-Cl_ = 1	ln*k* = ‒5.74 × $\sum \sigma_{o,\;m,\;p}^{+}$ − 23.90	*Q*^2^ = 0.825
	N_m-Cl_ = 2	ln*k* = ‒5.82 × $\sum \sigma_{o,\;m,\;p}^{+}$ − 22.11	F = 113
	N_m-Cl_ = 3	ln*k* = ‒5.81 × $\sum \sigma_{o,\;m,\;p}^{+}$ − 20.37	*p* < 0.01
	N_m-Cl_ = 4	ln*k* = ‒5.88 × $\sum \sigma_{o,\;m,\;p}^{+}$ − 18.53	[21]
PCDDs	N_m-Cl_ = 0	log*K*_OW_ = 4.60 × $\sum \sigma_{o,\;m,\;p}^{+}$ + 4.46	$\bar{{\Delta \log K}_{\mathrm{OW}}}$ = 0.45
	N_m-Cl_ = 1	log*K*_OW_ = 4.70 × $\sum \sigma_{o,\;m,\;p}^{+}$ + 2.97	*Q*^2^ = 0.954
	N_m-Cl_ = 2	log*K*_OW_ = 4.70 × $\sum \sigma_{o,\;m,\;p}^{+}$ + 1.48	F = 314
	N_m-Cl_ = 3	log*K*_OW_ = 4.69 × $\sum \sigma_{o,\;m,\;p}^{+}$ + 0.02	*p* < 0.01
	N_m-Cl_ = 4	log*K*_OW_ = 4.69 × $\sum \sigma_{o,\;m,\;p}^{+}-$ 1.45	[43] *
PCDDs	N_m-Cl_ = 0	−log*S*_W_ = 9.52 × $\sum \sigma_{o,\;m,\;p}^{+}$ + 4.78	$\bar{\Delta -{\log S}_{W}}$ = −0.92
	N_m-Cl_ = 1	−log*S*_W_ = 9.73 × $\sum \sigma_{o,\;m,\;p}^{+}$ + 1.69	*Q*^2^ = 0.981
	N_m-Cl_ = 2	−log*S*_W_ = 9.74 × $\sum \sigma_{o,\;m,\;p}^{+}$ − 1.38	F = 659
	N_m-Cl_ = 3	−log*S*_W_ = 9.72 × $\sum \sigma_{o,\;m,\;p}^{+}$ − 4.42	*p* < 0.01
	N_m-Cl_ = 4	−log*S*_W_ = 9.72 × $\sum \sigma_{o,\;m,\;p}^{+}$ − 7.46	[45] *

* The quantum-chemical descriptor model obtained from log*K*_OW_ = 0.03345 × *α* + 0.39092 and −log*S*_W_ = 0.0693 × *α* − 3.6425 [43,45].

## Supplementary Materials

The following are available online.
