# Supplementary material for: The Internal Relation between Quantum Chemical Descriptors and Empirical Constants of Polychlorinated Compounds
Source: Molecules. 2018 Nov 10;23(11):2935. doi: 10.3390/molecules23112935 (PMC6278375; doi:10.3390/molecules23112935)
Supplement: Supplementary file 1 [file molecules-23-02935-s001.pdf]

molecules

MDPI

Supplementary Materials

The internal relation between quantum chemical descriptors and empirical constants of polychlorinated biphenyls

Jiangchi Fei [1], Qiming Mao [2], Lu Peng [3], Tiantian Ye [4], Yuan Yang [2,*] and Shuang Luo [2,*]

1 Institute of Environmental Engineering, School of Metallurgy and Environment, Central South University, Changsha, China, 410083; E-mail: jack-fei@csu.edu.cn.
2 College of Resources and Environment, Hunan Agricultural University, Changsha, 410128, China; E-mail: qimingmao@126.com.
3 College of Biology and Environmental Sciences, Jishou University, Jishou, China, 416000; E-mail: abonluo@gmail.com.
4 South China Institute of Environmental Sciences, Ministry of Environmental Protection, Guangzhou, 510655, China; E-mail: yetiantian@scies.org.

* Correspondence: yangyuan@hunau.edu.cn (Y.Y.); luoshuang@csu.edu.cn or shuangluo@hunau.edu.cn (S.L.); Tel.: +86-151-1120-6841 (S.L.)

molecules

Text S1:

Hammett constant, σ, is a reflection of the electronic nature and position of substituent, as indicated below:

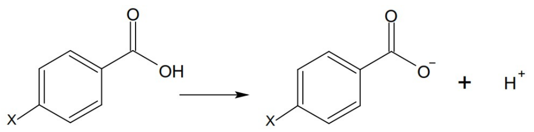

$$\log\left(\frac{k_{\text{X-Ar}}}{k_{\text{H-Ar}}}\right) = \sigma\rho \quad (1)$$

where X is the functional group substituted on aromatic compounds (Ar), k_X and k_H are the rate constants for substituted and unsubstituted benzene derivatives, respectively, and ρ is the reaction constant which depends on the type of reaction [1]. Both σ and ρ are obtained from a reference reaction, and can quantitatively describe chemical reactivities of substituents [1-3].

The σ^- is one for groups that stabilize negative charges via resonance, σ^- scale is based upon the ionization of *para*–substituted phenols [4]. The equation for σ^- is:

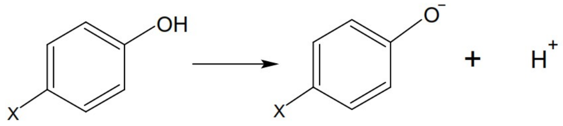

$$\log\left(\frac{k_{\text{X-C}_6\text{H}_4\text{OH}}}{k_{\text{H-C}_6\text{H}_4\text{OH}}}\right) = \sigma_X^-\rho$$

The σ^+ is one for groups that stabilized positive charges via resonance, based upon the heterolysis reaction of *para*-substituted cumyl chlorides (phenyldimethyl chloromethanes). The equation for σ^+ is:

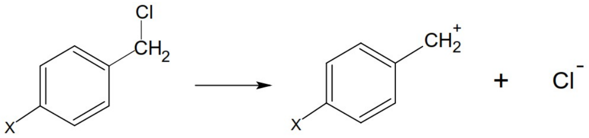

$$\log\left(\frac{k_{\text{X-C}_6\text{H}_4\text{CH}_2\text{Cl}}}{k_{\text{H-C}_6\text{H}_4\text{CH}_2\text{Cl}}}\right) = \sigma_X^+\rho$$

molecules

Table S1: The Hammett constants (σ, σ^-, σ^+) values

substituents	−Cl
σ_o	0.4
σ_m	0.37
σ_p	0.23
σ_o^+	0.073*
σ_m^+	0.4
σ_p^+	0.11
σ_o^-	0.19**
σ_m^-	0.37
σ_p^-	0.19

* The σ_o^+ values were calculated from the relationship $\sigma_o^+ = 0.66\sigma_p^+$, which accounts for the ortho effects. [5]

** σ_o^- values were sometimes obtained from the relationship $\sigma_o^- = \sigma_p^-$ when they are not available in literature. [4, 6]

molecules

Table S2: The nineteen molecular descriptors from quantum chemistry in this study.

descriptor	comment	unit
μ	molecular dipole moment	debye
EA	electron affinity	eV
E_{HOMO}	energy of the highest occupied molecular orbital	eV
E_{HOMO-1}	energy of the second HOMO	eV
E_{LUMO}	energy of the lowest unoccupied molecular orbital	eV
$E_{LUMO} - E_{HOMO}$	gap of E_{LUMO} and E_{HOMO}	eV
E_{LUMO+1}	energy of the second LUMO	eV
IP	ionization potential	eV
Q_{xx}	quadrupole moment tensor along the x axis	debye
Q_{yy}	quadrupole moment tensors along the y axis	debye
Q_{zz}	quadrupole moment tensors long the z axis	debye
S	softness, $S = 1/(IP - EA)$	eV^{-1}
α	mean polarizability of the molecule	$Bohr^3$
ζ	electronegativity, $\zeta = (IP + EA)/2$	eV
η	hardness, $\eta = (IP - EA)/2$	eV
ω	electrophilicity index, $\omega = \zeta^2/2\eta$	eV

Table S3: The structure of PCBs.

#	Name	structure	#	Name	structure	#	Name	structure
biphenyl	biphenyl	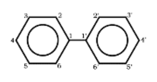	PCB18	2,2’,5-Trichlorobiphenyl	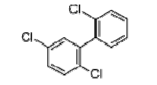	PCB36	3,3’,5-Trichlorobiphenyl	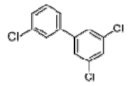
PCB1	2-Chlorobiphenyl	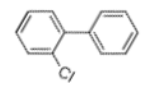	PCB19	2,2’,6-Trichlorobiphenyl	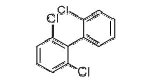	PCB37	3,4,4’-Trichlorobiphenyl	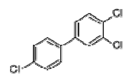
PCB2	3-Chlorobiphenyl	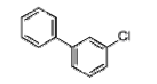	PCB20	2,3,3’-Trichlorobiphenyl	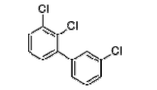	PCB38	3,4,5-Trichlorobiphenyl	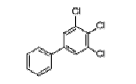
PCB3	4-Chlorobiphenyl	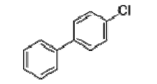	PCB21	2,3,4-Trichlorobiphenyl	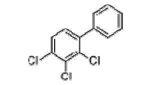	PCB39	3,4’,5-Trichlorobiphenyl	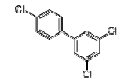
PCB4	2,2’-Dichlorobiphenyl	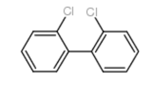	PCB22	2,3,4’-Trichlorobiphenyl	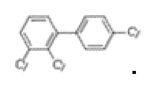	PCB40	2,2’,3,3’-Tetrachlorobiphenyl	
PCB5	2,3-Dichlorobiphenyl	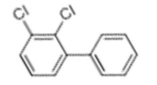	PCB23	2,3,5-Trichlorobiphenyl	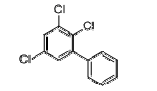	PCB41	2,2’,3,4-Tetrachlorobiphenyl	
PCB6	2,3’-Dichlorobiphenyl	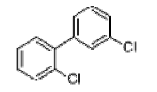	PCB24	2,3,6-Trichlorobiphenyl	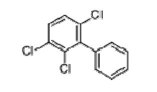	PCB42	2,2’,3,4’-Tetrachlorobiphenyl	
PCB7	2,4-Dichlorobiphenyl	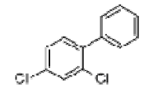	PCB25	2,3’,4-Trichlorobiphenyl	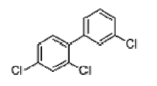	PCB43	2,2’,3,5-Tetrachlorobiphenyl	
PCB8	2,4’-Dichlorobiphenyl	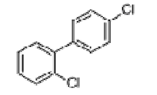	PCB26	2,3’,5-Trichlorobiphenyl	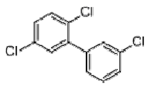	PCB44	2,2’,3,5’-Tetrachlorobiphenyl	
PCB9	2,5-Dichlorobiphenyl	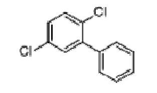	PCB27	2,3’,6-Trichlorobiphenyl	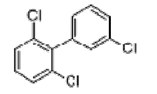	PCB45	2,2’,3,6-Tetrachlorobiphenyl	
PCB10	2,6-Dichlorobiphenyl	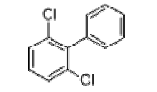	PCB28	2,4,4’-Trichlorobiphenyl	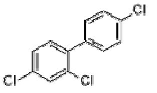	PCB46	2,2’,3,6’-Tetrachlorobiphenyl	
PCB11	3,3’-Dichlorobiphenyl	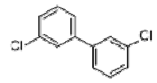	PCB29	2,4,5-Trichlorobiphenyl	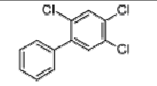	PCB47	2,2’,4,4’-Tetrachlorobiphenyl	
PCB12	3,4-Dichlorobiphenyl	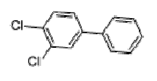	PCB30	2,4,6-Trichlorobiphenyl	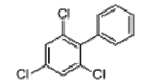	PCB48	2,2’,4,5-Tetrachlorobiphenyl	
PCB13	3,4’-Dichlorobiphenyl	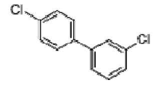	PCB31	2,4’,5-Trichlorobiphenyl	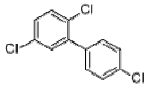	PCB49	2,2’,4,5’-Tetrachlorobiphenyl	
PCB14	3,5-Dichlorobiphenyl	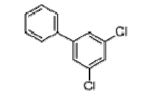	PCB32	2,4’,6-Trichlorobiphenyl	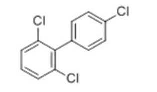	PCB50	2,2’,4,6-Tetrachlorobiphenyl	
PCB15	4,4’-Dichlorobiphenyl	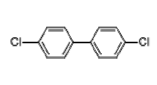	PCB33	2’,3,4-Trichlorobiphenyl	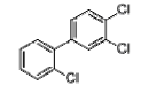	PCB51	2,2’,4,6’-Tetrachlorobiphenyl	
PCB16	2,2’,3-Trichlorobiphenyl	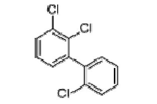	PCB34	2’,3,5-Trichlorobiphenyl	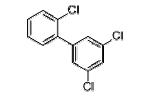	PCB52	2,2’,5,5’-Tetrachlorobiphenyl	
PCB17	2,2’,4-Trichlorobiphenyl	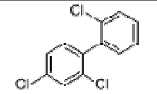	PCB35	3,3’,4-Trichlorobiphenyl	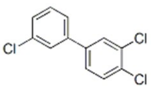	PCB53	2,2’,5,6’-Tetrachlorobiphenyl	

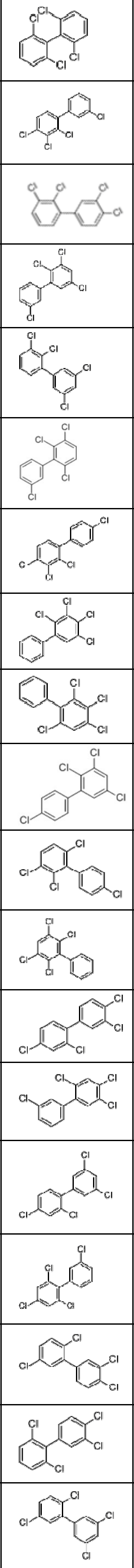
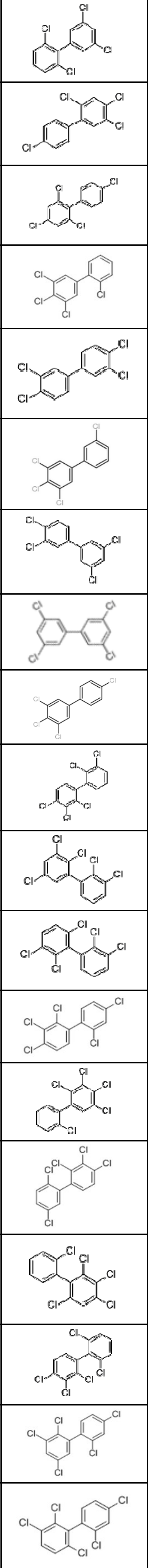
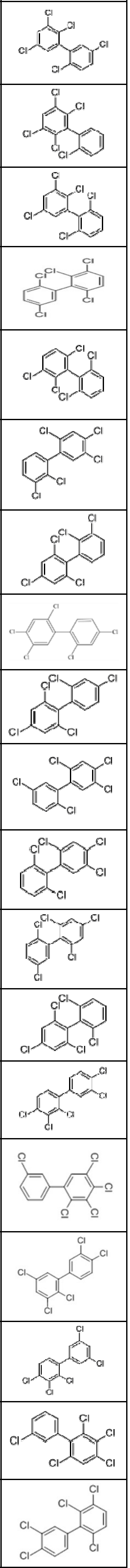

#	Name	structure	#	Name	structure	#	Name	structure
PCB54	2,2’,6,6’-Tetra chlorobiphenyl		PCB73	2,3’,5’,6-Tetrac hlorobiphenyl		PCB92	2,2’,3,5,5’-Penta chlorobiphenyl	
PCB55	2,3,3’,4-Tetrac hlorobiphenyl		PCB74	2,4,4’,5-Tetrach lorobiphenyl		PCB93	2,2’,3,5,6-Penta chlorobiphenyl	
PCB56	2,3,3’,4’-Tetra chlorobiphenyl		PCB75	2,4,4’,6-Tetrach lorobiphenyl		PCB94	2,2’,3,5,6’-Penta chlorobiphenyl	
PCB57	2,3,3’,5-Tetrac hlorobiphenyl		PCB76	2’,3,4,5-Tetrach lorobiphenyl		PCB95	2,2’,3,5’,6-Penta chlorobiphenyl	
PCB58	2,3,3’,5’-Tetra chlorobiphenyl		PCB77	3,3’,4,4’-Tetrac hlorobiphenyl		PCB96	2,2’,3,6,6’-Penta chlorobiphenyl	
PCB59	2,3,3’,6-Tetrac hlorobiphenyl		PCB78	3,3’,4,5-Tetrach lorobiphenyl		PCB97	2,2’,3’,4,5-Penta chlorobiphenyl	
PCB60	2,3,4,4’-Tetrac hlorobiphenyl		PCB79	3,3’,4,5’-Tetrac hlorobiphenyl		PCB98	2,2’,3’,4,6-Penta chlorobiphenyl	
PCB61	2,3,4,5-Tetrach lorobiphenyl		PCB80	3,3’,5,5’-Tetrac hlorobiphenyl		PCB99	2,2’,4,4’,5-Penta chlorobiphenyl	
PCB62	2,3,4,6-Tetrach lorobiphenyl		PCB81	3,4,4’,5-Tetrach lorobiphenyl		PCB100	2,2’,4,4’,6-Penta chlorobiphenyl	
PCB63	2,3,4’,5-Tetrac hlorobiphenyl		PCB82	2,2’,3,3’,4-Penta chlorobiphenyl		PCB101	2,2’,4,5,5’-Penta chlorobiphenyl	
PCB64	2,3,4’,6-Tetrac hlorobiphenyl		PCB83	2,2’,3,3’,5-Penta chlorobiphenyl		PCB102	2,2’,4,5,6’-Penta chlorobiphenyl	
PCB65	2,3,5,6-Tetrach lorobiphenyl		PCB84	2,2’,3,3’,6-Penta chlorobiphenyl		PCB103	2,2’,4,5’,6-Penta chlorobiphenyl	
PCB66	2,3’,4,4’-Tetra chlorobiphenyl		PCB85	2,2’,3,4,4’-Penta chlorobiphenyl		PCB104	2,2’,4,6,6’-Penta chlorobiphenyl	
PCB67	2,3’,4,5-Tetrac hlorobiphenyl		PCB86	2,2’,3,4,5-Penta chlorobiphenyl		PCB105	2,3,3’,4,4’-Penta chlorobiphenyl	
PCB68	2,3’,4,5’-Tetra chlorobiphenyl		PCB87	2,2’,3,4,5’-Penta chlorobiphenyl		PCB106	2,3,3’,4,5-Penta chlorobiphenyl	
PCB69	2,3’,4,6-Tetrac hlorobiphenyl		PCB88	2,2’,3,4,6-Penta chlorobiphenyl		PCB107	2,3,3’,4,5’-Penta chlorobiphenyl	
PCB70	2,3’,4’,5-Tetra chlorobiphenyl		PCB89	2,2’,3,4,6’-Penta chlorobiphenyl		PCB108	2,3,3’,4,6-Penta chlorobiphenyl	
PCB71	2,3’,4’,6-Tetra chlorobiphenyl		PCB90	2,2’,3,4’,5-Penta chlorobiphenyl		PCB109	2,3,3’,4’,5-Penta chlorobiphenyl	
PCB72	2,3’,5,5’-Tetra chlorobiphenyl		PCB91	2,2’,3,4’,6-Penta chlorobiphenyl		PCB110	2,3,3’,4’,6-Penta chlorobiphenyl	

#	Name	structure	#	Name	structure	#	Name	structure
PCB111	2,3,3',5,5'-Pentachlorobiphenyl		PCB130	2,2',3,3',4,5'-Hexachlorobiphenyl		PCB149	2,2',3,4',5',6-Hexachlorobiphenyl	
PCB112	2,3,3',5,6-Pentachlorobiphenyl		PCB131	2,2',3,3',4,6-Hexachlorobiphenyl		PCB150	2,2',3,4',6,6'-Hexachlorobiphenyl	
PCB113	2,3,3',5',6-Pentachlorobiphenyl		PCB132	2,2',3,3',4,6'-Hexachlorobiphenyl		PCB151	2,2',3,5,5',6-Hexachlorobiphenyl	
PCB114	2,3,4,4',5-Pentachlorobiphenyl		PCB133	2,2',3,3',5,5'-Hexachlorobiphenyl		PCB152	2,2',3,5,6,6'-Hexachlorobiphenyl	
PCB115	2,3,4,4',6-Pentachlorobiphenyl		PCB134	2,2',3,3',5,6-Hexachlorobiphenyl		PCB153	2,2',4,4',5,5'-Hexachlorobiphenyl	
PCB116	2,3,4,5,6-Pentachlorobiphenyl		PCB135	2,2',3,3',5,6'-Hexachlorobiphenyl		PCB154	2,2',4,4',5,6'-Hexachlorobiphenyl	
PCB117	2,3,4',5,6-Pentachlorobiphenyl		PCB136	2,2',3,3',6,6'-Hexachlorobiphenyl		PCB155	2,2',4,4',6,6'-Hexachlorobiphenyl	
PCB118	2,3',4,4',5-Pentachlorobiphenyl		PCB137	2,2',3,4,4',5-Hexachlorobiphenyl		PCB156	2,3,3',4,4',5-Hexachlorobiphenyl	
PCB119	2,3',4,4',6-Pentachlorobiphenyl		PCB138	2,2',3,4,4',5'-Hexachlorobiphenyl		PCB157	2,3,3',4,4',5'-Hexachlorobiphenyl	
PCB120	2,3',4,5,5'-Pentachlorobiphenyl		PCB139	2,2',3,4,4',6-Hexachlorobiphenyl		PCB158	2,3,3',4,4',6-Hexachlorobiphenyl	
PCB121	2,3',4,5',6-Pentachlorobiphenyl		PCB140	2,2',3,4,4',6'-Hexachlorobiphenyl		PCB159	2,3,3',4,5,5'-Hexachlorobiphenyl	
PCB122	2',3,3',4,5-Pentachlorobiphenyl		PCB141	2,2',3,4,5,5'-Hexachlorobiphenyl		PCB160	2,3,3',4,5,6-Hexachlorobiphenyl	
PCB123	2',3,4,4',5-Pentachlorobiphenyl		PCB142	2,2',3,4,5,6-Hexachlorobiphenyl		PCB161	2,3,3',4,5',6-Hexachlorobiphenyl	
PCB124	2',3,4,5,5'-Pentachlorobiphenyl		PCB143	2,2',3,4,5,6'-Hexachlorobiphenyl		PCB162	2,3,3',4',5,5'-Hexachlorobiphenyl	
PCB125	2',3,4,5,6'-Pentachlorobiphenyl		PCB144	2,2',3,4,5',6-Hexachlorobiphenyl		PCB163	2,3,3',4',5,6-Hexachlorobiphenyl	
PCB126	3,3',4,4',5-Pentachlorobiphenyl		PCB145	2,2',3,4,6,6'-Hexachlorobiphenyl		PCB164	2,3,3',4',5',6-Hexachlorobiphenyl	
PCB127	3,3',4,5,5'-Pentachlorobiphenyl		PCB146	2,2',3,4',5,5'-Hexachlorobiphenyl		PCB165	2,3,3',5,5',6-Hexachlorobiphenyl	
PCB128	2,2',3,3',4,4'-Hexachlorobiphenyl		PCB147	2,2',3,4',5,6-Hexachlorobiphenyl		PCB166	2,3,4,4',5,6-Hexachlorobiphenyl	
PCB129	2,2',3,3',4,5-Hexachlorobiphenyl		PCB148	2,2',3,4',5,6'-Hexachlorobiphenyl		PCB167	2,3',4,4',5,5'-Hexachlorobiphenyl	

#	Name	structure	#	Name	structure	#	Name	structure
PCB168	2,3’,4,4’,5’,6-Hexachlorobiphenyl	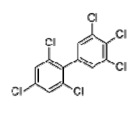	CB182	2,2’,3,4,4’,5,6’-Heptachlorobiphenyl	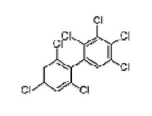	PCB196	2,2’,3,3’,4,4’,5,6’-Octachlorobiphenyl	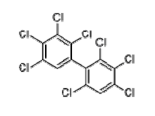
PCB169	3,3’,4,4’,5,5’-Hexachlorobiphenyl	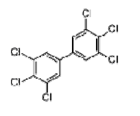	PCB183	2,2’,3,4,4’,5’,6-Heptachlorobiphenyl	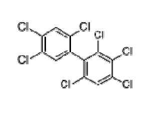	PCB197	2,2’,3,3’,4,4’,6,6’-Octachlorobiphenyl	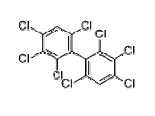
PCB170	2,2’,3,3’,4,4’,5-Heptachlorobiphenyl	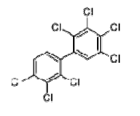	PCB184	2,2’,3,4,4’,6,6’-Heptachlorobiphenyl	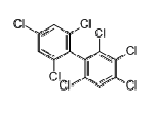	PCB198	2,2’,3,3’,4,5,5’,6-Octachlorobiphenyl	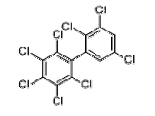
PCB171	2,2’,3,3’,4,4’,6-Heptachlorobiphenyl	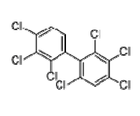	PCB185	2,2’,3,4,5,5’,6-Heptachlorobiphenyl	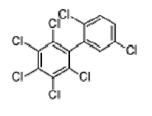	PCB199	2,2’,3,3’,4,5,5’,6’-Octachlorobiphenyl	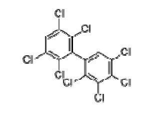
PCB172	2,2’,3,3’,4,5,5’-Heptachlorobiphenyl	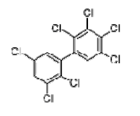	PCB186	2,2’,3,4,5,6,6’-Heptachlorobiphenyl	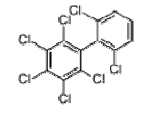	PCB200	2,2’,3,3’,4,5,6,6’-Octachlorobiphenyl	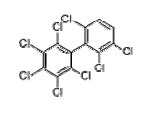
PCB173	2,2’,3,3’,4,5,6-Heptachlorobiphenyl	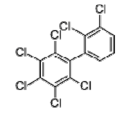	PCB187	2,2’,3,4’,5,5’,6-Heptachlorobiphenyl	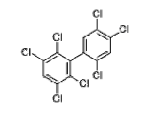	PCB201	2,2’,3,3’,4,5’,6,6’-Octachlorobiphenyl	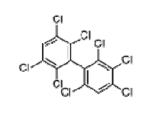
PCB174	2,2’,3,3’,4,5,6’-Heptachlorobiphenyl	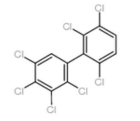	PCB188	2,2’,3,4’,5,6,6’-Heptachlorobiphenyl	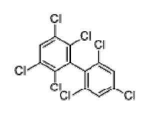	PCB202	2,2’,3,3’,5,5’,6,6’-Octachlorobiphenyl	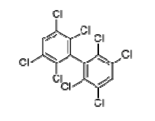
PCB175	2,2’,3,3’,4,5’,6-Heptachlorobiphenyl	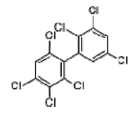	PCB189	2,3,3’,4,4’,5,5’-Heptachlorobiphenyl	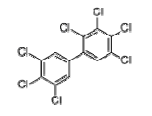	PCB203	2,2’,3,4,4’,5,5’,6-Octachlorobiphenyl	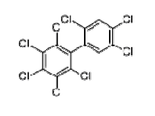
PCB176	2,2’,3,3’,4,6,6’-Heptachlorobiphenyl	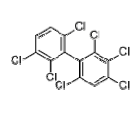	PCB190	2,3,3’,4,4’,5,6-Heptachlorobiphenyl	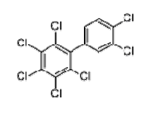	PCB204	2,2’,3,4,4’,5,6,6’-Octachlorobiphenyl	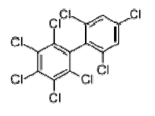
PCB177	2,2’,3,3’,4’,5,6-Heptachlorobiphenyl	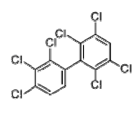	PCB191	2,3,3’,4,4’,5’,6-Heptachlorobiphenyl	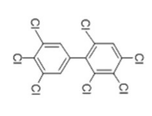	PCB205	2,3,3’,4,4’,5,5’,6-Octachlorobiphenyl	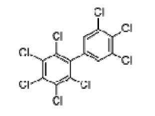
PCB178	2,2’,3,3’,5,5’,6-Heptachlorobiphenyl	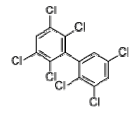	PCB192	2,3,3’,4,5,5’,6-Heptachlorobiphenyl	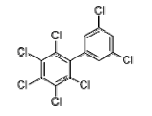	PCB206	2,2’,3,3’,4,4’,5,5’,6-Nonachlorobiphenyl	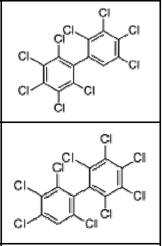
PCB179	2,2’,3,3’,5,6,6’-Heptachlorobiphenyl	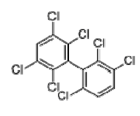	PCB193	2,3,3’,4’,5,5’,6-Heptachlorobiphenyl	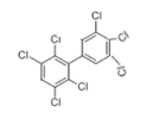	PCB207	2,2’,3,3’,4,4’,5,6,6’-Nonachlorobiphenyl	
PCB180	2,2’,3,4,4’,5,5’-Heptachlorobiphenyl	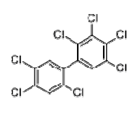	PCB194	2,2’,3,3’,4,4’,5,5’-Octachlorobiphenyl	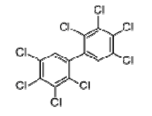	PCB208	2,2’,3,3’,4,5,5’,6,6’-Nonachlorobiphenyl	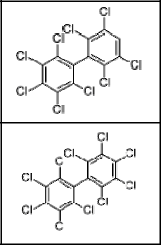
PCB181	2,2’,3,4,4’,5,6-Heptachlorobiphenyl	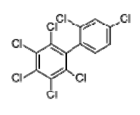	PCB195	2,2’,3,3’,4,4’,5,6-Octachlorobiphenyl	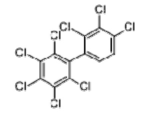	PCB209	2,2’,3,3’,4,4’,5,5’,6,6’-Decachlorobiphenyl	

Table S4: The structure of PCDDs.

#	Name	structure	#	Name	structure	#	Name	structure
Dibenzo-1,4-dioxin	Dibenzo-1,4-dioxin	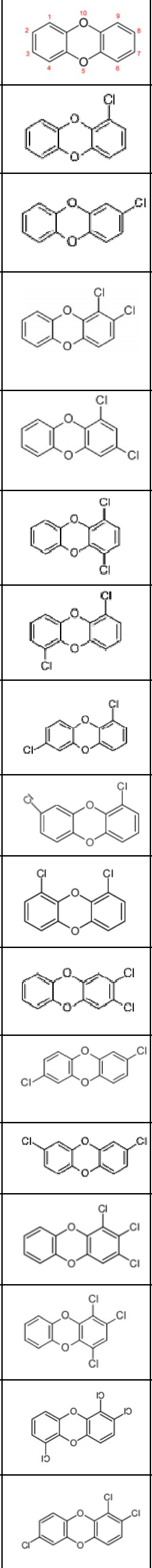	PCDD18	1,2,9-Trichlorodibenzodioxin	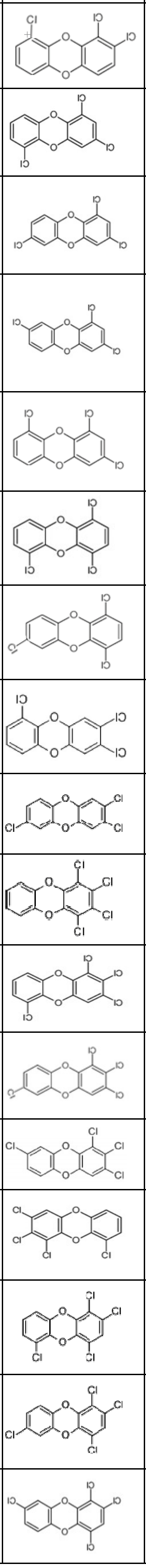	PCDD36	1,2,6,7-Tetrachlorodibenzo-p-dioxin	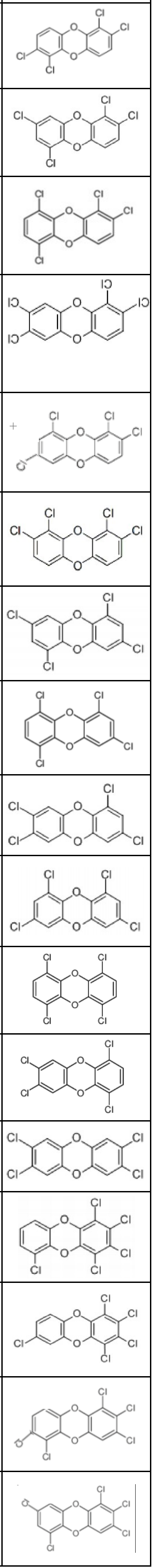
PCDD1	1-Chlorodibenzo-p-dioxin		PCDD19	1,3,6-Trichlorodibenzodioxin		PCDD37	1,2,6,8-Tetrachlorodibenzo-p-dioxin	
PCDD2	2-Chlorodibenzo-p-dioxin		PCDD20	1,3,7-Trichlorodibenzodioxin		PCDD38	1,2,6,9-Tetrachlorodibenzo-p-dioxin	
PCDD3	1,2-Dichlorodibenzo-p-dioxin		PCDD21	1,3,8-Trichlorodibenzodioxin		PCDD39	1,2,7,8-Tetrachlorodibenzo-p-dioxin	
PCDD4	1,3-Dichlorodibenzo-p-dioxin		PCDD22	1,3,9-Trichlorodibenzodioxin		PCDD40	1,2,7,9-Tetrachlorodibenzodioxin	
PCDD5	1,4-Dichlorodibenzo-p-dioxin		PCDD23	1,4,6-trichlorodibenzo-p-dioxin		PCDD41	1,2,8,9-Tetrachlorodibenzo-p-dioxin	
PCDD6	1,6-Dichlorodibenzo-p-dioxin		PCDD24	1,4,7-trichlorodibenzo-p-dioxin		PCDD42	1,3,6,8-Tetrachlorobenzo-p-dioxin	
PCDD7	1,7-Dichlorodibenzo-p-dioxin		PCDD25	2,3,6-Trichlorodibenzo-p-dioxin		PCDD43	1,3,6,9-Tetrachlorobenzo-p-dioxin	
PCDD8	1,8-Dichlorodibenzo-p-dioxin		PCDD26	2,3,7-Trichlorodibenzo-p-dioxin		PCDD44	1,3,7,8-Tetrachlorobenzo-p-dioxin	
PCDD9	1,9-dichlorodibenzo-p-dioxin		PCDD27	1,2,3,4-Tetrachlorodibenzo-p-dioxin		PCDD45	1,3,7,9-Tetrachlorodibenzo-p-dioxin	
PCDD10	2,3-Dichlorodibenzo-p-dioxin		PCDD28	1,2,3,6-Tetrachlorodibenzo-p-dioxin		PCDD46	1,4,6,9-tetrachlorodibenzo-p-dioxin	
PCDD11	2,7-Dichlorodibenzo-p-dioxin		PCDD29	1,2,3,7-Tetrachlorodibenzo-p-dioxin		PCDD47	1,4,7,8-tetrachlorodibenzo-p-dioxin	
PCDD12	2,8-Dichlorodibenzo-p-dioxin		PCDD30	1,2,3,8-Tetrachlorodibenzo-p-dioxin		PCDD48	2,3,7,8-tetrachlorodibenzo-p-dioxin	
PCDD13	1,2,3-Trichlorodibenzo-p-dioxin		PCDD31	1,2,3,9-Tetrachlorodibenzo-p-dioxin		PCDD49	Pentachlorodibenzo-p-dioxin	
PCDD14	1,2,4-Trichlorodibenzo-p-dioxin		PCDD32	1,2,4,6-Tetrachlorodibenzo-p-dioxin		PCDD50	1,2,3,4,7-Pentachlorodibenzo-p-dioxin	
PCDD15	1,2,6-Trichlorodibenzodioxin;		PCDD33	1,2,4,7-Tetrachlorodibenzo-p-dioxin		PCDD51	1,2,3,6,7-Pentachlorodibenzo-p-dioxin	
PCDD16	1,2,7-trichlorodibenzo-p-dioxin		PCDD34	1,2,4,8-Tetrachlorodibenzodioxin		PCDD52	1,2,3,6,8-Pentachlorodibenzo-p-dioxin	

PCDD17	1,2,8-Trichlorodibenzodioxin	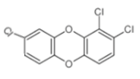	PCDD35	1,2,4,9-Tetrachlorodibenzodioxin	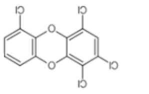	PCDD53	1,2,3,6,9-Pentachlorodibenzo-p-dioxin	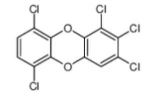

#	**Name**	**structure**	#	**Name**	**structure**	#	**Name**	**structure**
PCDD54	1,2,3,7,8-Pentachlorodibenzo-p-dioxin	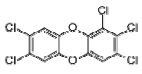	PCDD62	1,2,4,8,9-Pentachlorodibenzo-p-dioxin	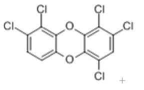	PCDD70	1,2,3,7,8,9-Hexachlorodibenzo-p-dioxin	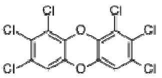
PCDD55	1,2,3,7,9-Pentachlorodibenzo-p-dioxin	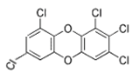	PCDD63	1,2,3,4,6,7-Hexachlorodibenzo-p-dioxin	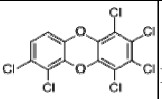	PCDD71	1,2,4,6,7,9-Hexachlorodibenzo-p-dioxin	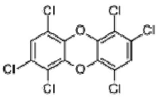
PCDD56	1,2,3,8,9-Pentachlorodibenzo-p-dioxin	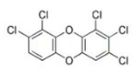	PCDD64	1,2,3,4,6,8-Hexachlorodibenzo-p-dioxin	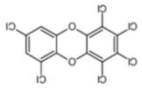	PCDD72	1,2,4,6,8,9-Hexachlorodibenzo-p-dioxin	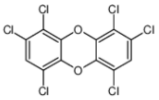
PCDD57	1,2,4,6,7-Pentachlorodibenzodioxin	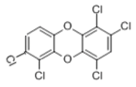	PCDD65	1,2,3,4,6,9-Hexachlorodibenzo-p-dioxin	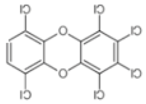	PCDD73	1,2,3,4,6,7,8-Heptachlorodibenzo-p-dioxin	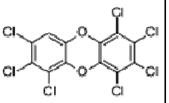
PCDD58	1,2,4,6,8-Pentachlorodibenzo-p-dioxin	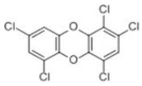	PCDD66	1,2,3,4,7,8-Hexachlorodibenzo-p-dioxin	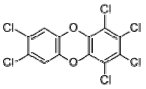	PCDD74	1,2,3,4,6,7,9-Heptachlorodibenzo-p-dioxin	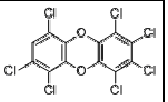
PCDD59	1,2,4,6,9-Pentachlorodibenzo-p-dioxin	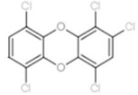	PCDD67	1,2,3,6,7,8-Hexachlorodibenzo-p-dioxin	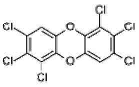	PCDD75	1,2,3,4,6,7,8,9-Octachlorodibenzo-p-dioxin	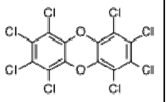
PCDD60	1,2,4,7,8-Pentachlorodibenzo-p-dioxin	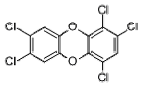	PCDD68	1,2,3,6,7,9-Hexachlorodibenzo-p-dioxin	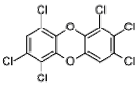			
PCDD61	1,2,4,7,9-Pentachlorodibenzo-p-dioxin	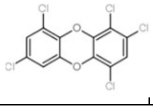	PCDD69	1,2,3,6,8,9-hexachlorodibenzo-p-dioxin	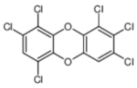			

molecules

MDPI

Table S5: The structure of PCNs.

#	Name	structure	#	Name	structure	#	Name	structure
naphthalene	naphthalene	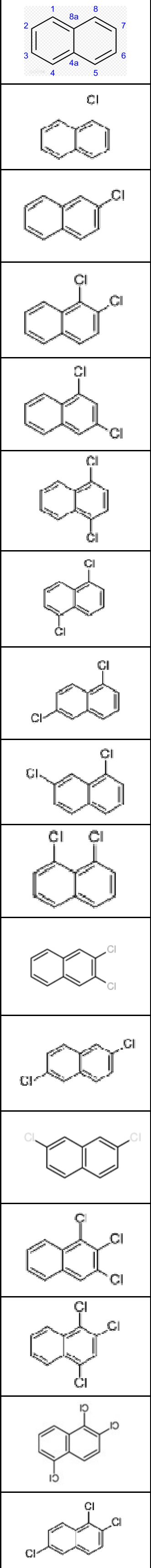	PCN17	1,2,7-Trichloronaphthalene	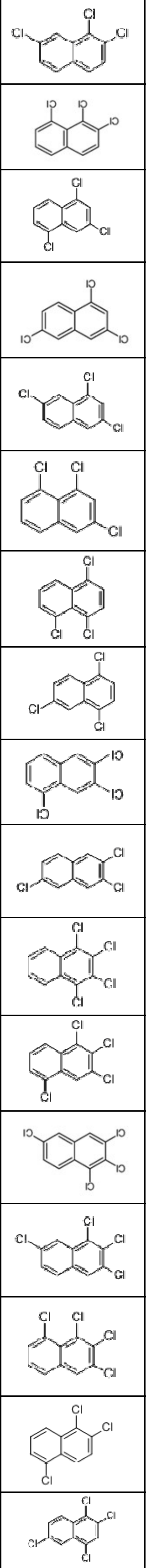	PCN34	1,2,4,7-Tetrachloronaphthalene	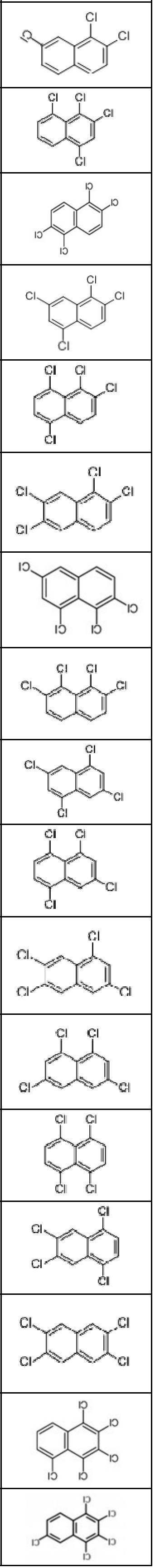
PCN1	1-Chloronaphthalene		PCN18	1,2,8-Trichloronaphthalene		PCN35	1,2,4,8-Tetrachloronaphthalene	
PCN2	2-Chloronaphthalene		PCN19	1,3,5-Trichloronaphthalene		PCN36	1,2,5,6-Tetrachloronaphthalene	
PCN3	1,2-Dichloronaphthalene		PCN20	1,3,6-Trichloronaphthalene		PCN37	1,2,5,7-Tetrachloronaphthalene	
PCN4	1,3-Dichloronaphthalene		PCN21	1,3,7-Trichloronaphthalene		PCN38	1,2,5,8-Tetrachloronaphthalene	
PCN5	1,4-Dichloronaphthalene		PCN22	1,3,8-Trichloronaphthalene		PCN39	1,2,6,7-Tetrachloronaphthalene	
PCN6	1,5-Dichloronaphthalene		PCN23	1,4,5-Trichloronaphthalene		PCN40	1,2,6,8-Tetrachloronaphthalene	
PCN7	1,6-Dichloronaphthalene		PCN24	1,4,6-Trichloronaphthalene		PCN41	1,2,7,8-Tetrachloronaphthalene	
PCN8	1,7-Dichloronaphthalene		PCN25	1,6,7-Trichloronaphthalene		PCN42	1,3,5,7-Tetrachloronaphthalene	
PCN9	1,8-Dichloronaphthalene		PCN26	2,3,6-Trichloronaphthalene		PCN43	1,3,5,8-Tetrachloronaphthalene	
PCN10	2,3-Dichloronaphthalene		PCN27	1,2,3,4-Tetrachloronaphthalene		PCN44	1,3,6,7-Tetrachloronaphthalene	
PCN11	2,6-Dichloronaphthalene		PCN28	1,2,3,5-Tetrachloronaphthalene		PCN45	1,3,6,8-Tetrachloronaphthalene	
PCN12	2,7-Dichloronaphthalene		PCN29	1,2,3,6-Tetrachloronaphthalene		PCN46	1,4,5,8-Tetrachloronaphthalene	
PCN13	trichloro-Naphthalene		PCN30	1,2,3,7-Tetrachloronaphthalene		PCN47	1,4,6,7-Tetrachloronaphthalene	
PCN14	1,2,4-Trichloronaphthalene		PCN31	1,2,3,8-Tetrachloronaphthalene		PCN48	2,3,6,7-Tetrachloronaphthalene	
PCN15	1,2,5-Trichloronaphthalene		PCN32	1,2,4,5-Tetrachloronaphthalene		PCN49	1,2,3,4,5-pentachloronaphthalene	
PCN16	1,2,6-Trichloronaphthalene		PCN33	1,2,4,6-Tetrachloronaphthalene		PCN50	1,2,3,4,6-pentachloronaphthalene	

**#**	**Name**	**structure**	**#**	**Name**	**structure**	**#**	**Name**	**structure**
PCN51	1,2,3,5,6-Pentachloronaphthalene	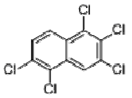	PCN60	1,2,4,6,7-Pentachloronaphthalene	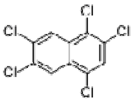	PCN69	1,2,3,5,7,8-Hexachloronaphthalene	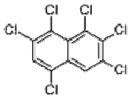
PCN52	1,2,3,5,7-Pentachloronaphthalene	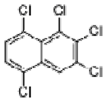	PCN61	1,2,4,6,8-Pentachloronaphthalene	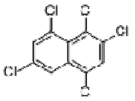	PCN70	1,2,3,6,7,8-Hexachloronaphthalene	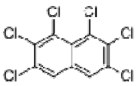
PCN53	1,2,3,5,8-Pentachloronaphthalene	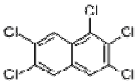	PCN62	1,2,4,7,8-Pentachloronaphthalene	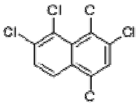	PCN71	Naphthalene,1,2,4,5,6,7-hexachloro-	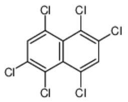
PCN54	1,2,3,6,7-Pentachloronaphthalene	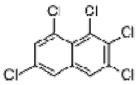	PCN63	1,2,3,4,5,6-Hexachloronaphthalene	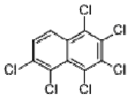	PCN72	1,2,4,5,7,8-Hexachloronaphthalene	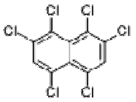
PCN55	1,2,3,6,8-Pentachloronaphthalene	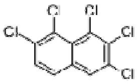	PCN64	1,2,3,4,5,7-Hexachloronaphthalene	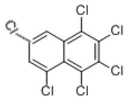	PCN73	1,2,3,4,5,6,7-Heptachloronaphthalene	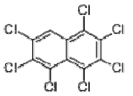
PCN56	1,2,3,7,8-Pentachloronaphthalene	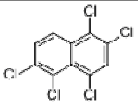	PCN65	1,2,3,4,5,8-Hexachloronaphthalene	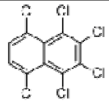	PCN74	1,2,3,4,5,6,8-Heptachloronaphthalene	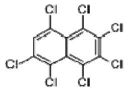
PCN57	1,2,4,5,6-Pentachloronaphthalene	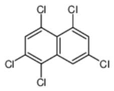	PCN66	1,2,3,4,6,7-Hexachloronaphthalene	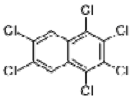	PCN75	1,2,3,4,5,6,7,8-Octachloronaphthalene	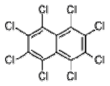
PCN58	1,2,4,5,7-Pentachloronaphthalene	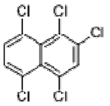	PCN67	1,2,3,5,6,7-Hexachloronaphthalene	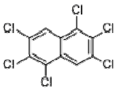			
PCN59	1,2,4,5,8-Pentachloronaphthalene	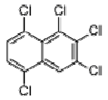	PCN68	1,2,3,5,6,8-Hexachloronaphthalene	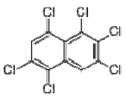			

molecules

Table S6: Comparison of lnk values of •OH oxidation of PCBs congeners (test set compounds) between observed and predicted values.

PCB conger	N_{m_cl}	observed ln$k$ [a]	predicted ln$k$	Δln$k$	PCB conger	N_{m_cl}	observed ln$k$ [a]	predicted ln$k$	Δln$k$
PCB0	0	-25.61	-25.86	0.253	PCB13	1	-26.89	-27.00	0.111
PCB 1	0	-26.60	-26.29	-0.306	PCB14	2	-26.57	-26.93	0.361
PCB2	1	-26.02	-26.44	0.416	PCB15	0	-27.10	-27.10	-0.002
PCB3	0	-26.30	-26.52	0.226	PCB20	2	-27.37	-27.22	-0.149
PCB4	0	-26.85	-26.57	-0.287	PCB28	0	-27.54	-27.41	-0.129
PCB5	1	-27.04	-26.75	-0.294	PCB29	1	-27.37	-27.34	-0.033
PCB6	1	-26.68	-26.76	0.082	PCB31	1	-27.41	-27.34	-0.070
PCB7	0	-26.71	-26.85	0.133	PCB33	1	-27.63	-27.29	-0.338
PCB8	0	-27.18	-26.83	-0.347	PCB44	2	-27.77	-27.53	-0.249
PCB9	1	-27.04	-26.79	-0.249	PCB47	0	-27.48	-27.64	0.166
PCB10	0	-27.04	-26.61	-0.430	PCB95	2	-27.99	-27.93	-0.059
PCB11	2	-26.64	-26.90	0.265	PCB110	2	-28.14	-28.08	-0.066
PCB12	1	-27.04	-26.99	-0.056	PCB116	2	-27.74	-28.12	0.381

a. The observed lnk values obtained from Anderson [7], Atkinson[8, 9] and Kwok [10] studies.

molecules MDPI

Table S7: Comparison of log*K*ow and log*S*w of PCDDs (test set compounds) between observed and predicted values.

PCDD congener	N_{m_cl}	observed log*K*ow [a]	predicted log*K*ow	$\Delta logK_{ow}$	observed log*S*w [b]	predicted log*S*w	Δ-logS_w
PCDD0	0	4.30	4.46	-0.157	-5.34	-4.78	-0.558
PCDD1	0	5.05	4.79	0.259	-5.72	-5.47	-0.247
PCDD2	1	5.00	4.84	0.156	-5.90	-5.58	-0.317
PCDD10	2	5.60	5.24	0.360	-7.23	-6.40	-0.827
PCDD11	2	5.75	5.24	0.510	-7.83	-6.40	-1.427
PBDD12	2	5.60	5.24	0.360	-7.18	-6.40	-0.777
PCDD14	1	6.35	5.53	0.824	-7.53	-6.99	-0.534
PCDD27	2	6.48	5.92	0.558	-8.71	-7.82	-0.894
PCDD29	3	6.48	5.99	0.492	-8.88	-7.95	-0.927
PCDD33	2	6.20	5.92	0.278	/	/	/
PCDD42	2	6.29	5.92	0.368	-9.00	-7.82	-1.184
PCDD48	4	6.42	6.05	0.365	-9.01	-8.09	-0.919
PCDD50	3	6.64	6.33	0.311	-9.48	-8.66	-0.821
PCDD54	4	6.60	6.40	0.205	/	/	/
PCDD66	4	7.80	6.74	1.064	-10.95	-9.50	-1.448
PCDD73	4	8.00	7.08	0.924	-11.25	-10.20	-1.042
PCDD75	4	8.20	7.42	0.783	-12.79	-10.91	-1.877
		$\overline{\Delta logK_{ow}}$		0.451	$\overline{\Delta\text{-}logS_w}$		−0.920

a. The observed log*K*ow values obtained from [11, 12]; b. The observed log*S*w values obtained from [13]

molecules MDPI

Table S8: The fitted linear equations of $\sum\sigma^{+}_{o,\,m,\,p}$ with $Q_{xx/yy/zz}$, α and E_{HOMO} for PCDDs congeners.

#	$\sum\sigma^{+}_{o,\,m,\,p}$ range	Q_{xx}= A × $\sum\sigma^{+}_{o,\,m,\,p}$+ B			Q_{yy}= A × $\sum\sigma^{+}_{o,\,m,\,p}$+ B			Q_{zz}= A × $\sum\sigma^{+}_{o,\,m,\,p}$+ B		
		**A**	**B**	**R^2**	**A**	**B**	**R^2**	**A**	**B**	**R^2**
$N_{m\text{-}Cl}$ =0	0~0.29	-151.43	-74.87	0.712	-232.70	-66.96	0.848	-161.95	-82.22	0.778
$N_{m\text{-}Cl}$ =1	0.4~0.69	-157.11	-26.70	0.868	-225.69	7.46	0.957	-162.95	-31.75	0.999
$N_{m\text{-}Cl}$ =2	0.8~1.09	-158.43	17.68	0.778	-201.56	61.44	0.878	-169.24	27.44	0.958
$N_{m\text{-}Cl}$ =3	1.2~1.49	-126.98	16.30	0.969	-223.37	159.27	0.977	-161.87	73.73	0.999
$N_{m\text{-}Cl}$ =4	1.6~2.89	-117.85	39.84	0.513	-217.60	219.15	0.820	-161.88	124.10	0.825

#	$\sum\sigma^{+}_{o,\,m,\,p}$ range	α = A × $\sum\sigma^{+}_{o,\,m,\,p}$+ B			E_{HOMO} = A × $\sum\sigma^{+}_{o,\,m,\,p}$+ B		
		**A**	**B**	**R^2**	**B**	**A**	**R^2**
$N_{m\text{-}Cl}$ =0	0~0.29	137.38	121.56	0.999	-3.38	-5.63	0.999
$N_{m\text{-}Cl}$ =1	0.4~0.69	140.41	76.96	0.998	-3.01	-4.68	0.992
$N_{m\text{-}Cl}$ =2	0.8~1.09	140.49	32.56	0.998	-2.68	-3.97	0.982
$N_{m\text{-}Cl}$ =3	1.2~1.49	140.26	-11.17	0.999	-2.40	-3.43	0.989
$N_{m\text{-}Cl}$ =4	1.6~2.89	140.23	-55.05	0.999	-2.14	-3.06	0.999

Table S9: The fitted linear equations of $\sum\sigma^+_{o,\,m,\,p}$ with $Q_{xx/yy/zz}$, α and E_{HOMO} for PCNs congeners.

#	$\sum\sigma^+_{o,\,m,\,p}$ range	Q_{xx}= A × $\sum\sigma^+_{o,\,m,\,p}$+ B			Q_{yy}= A × $\sum\sigma^+_{o,\,m,\,p}$+ B			Q_{zz}= A × $\sum\sigma^+_{o,\,m,\,p}$+ B		
		A	B	R^2			A	B	R^2	
$N_{m\text{-}Cl}$ =0	0~0.29	-240.02	-49.66	0.987	-216.10	-45.02	0.887	-162.41	-63.93	0.998
$N_{m\text{-}Cl}$ =1	0.4~0.69	-219.32	24.56	0.950	-182.29	10.34	0.941	-162.11	-10.85	0.999
$N_{m\text{-}Cl}$ =2	0.8~1.09	-164.29	42.91	0.883	-205.62	90.70	0.943	-162.10	42.65	0.970
$N_{m\text{-}Cl}$ =3	1.2~1.49	-139.30	58.61	0.982	-215.12	171.84	0.970	-162.36	95.65	0.999
$N_{m\text{-}Cl}$ =4	1.6~2.89	-114.93	49.67	0.782	-210.79	236.67	0.996	-162.75	149.49	0.997

#	$\sum\sigma^+_{o,\,m,\,p}$ range	α = A × $\sum\sigma^+_{o,\,m,\,p}$+ B			E_{HOMO} = A × $\sum\sigma^+_{o,\,m,\,p}$+ B		
		A	B	R^2	B	A	R^2
$N_{m\text{-}Cl}$ =0	0~0.29	144.24	94.35	0.997	-1.54	-6.33	0.674
$N_{m\text{-}Cl}$ =1	0.4~0.69	146.42	46.57	0.996	-1.32	-6.04	0.677
$N_{m\text{-}Cl}$ =2	0.8~1.09	147.19	-0.66	0.995	-0.97	-6.02	0.576
$N_{m\text{-}Cl}$ =3	1.2~1.49	146.79	-46.67	0.997	-0.65	-6.24	0.466
$N_{m\text{-}Cl}$ =4	1.6~2.89	146.54	-92.20	0.998	-0.43	-6.51	0.438

Molecules **2018**, *23*, 2935; doi: 10.3390/molecules23112935 www.mdpi.com/journal/molecules

molecules

MDPI

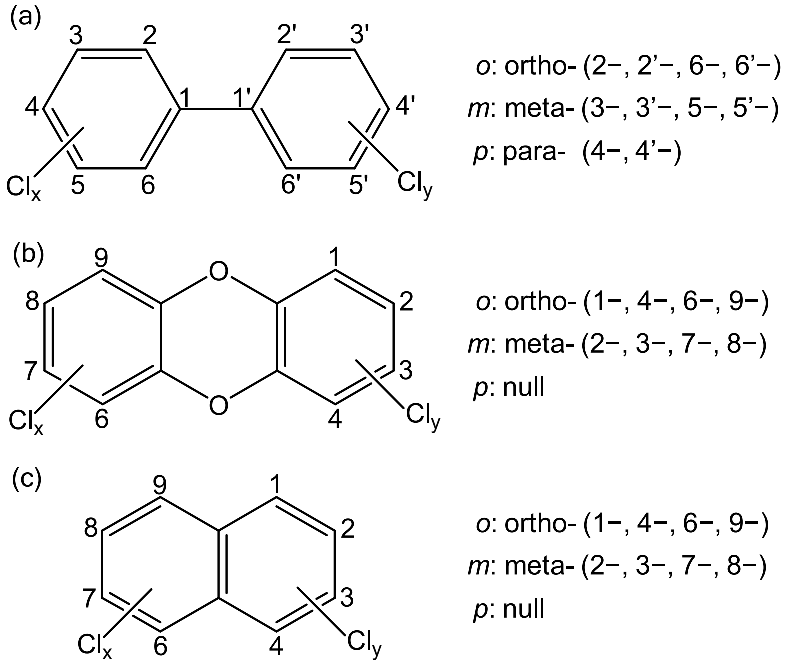

Figure S1: General molecular structures of the PCBs (a), PCDDs (b) and PCNs (c).

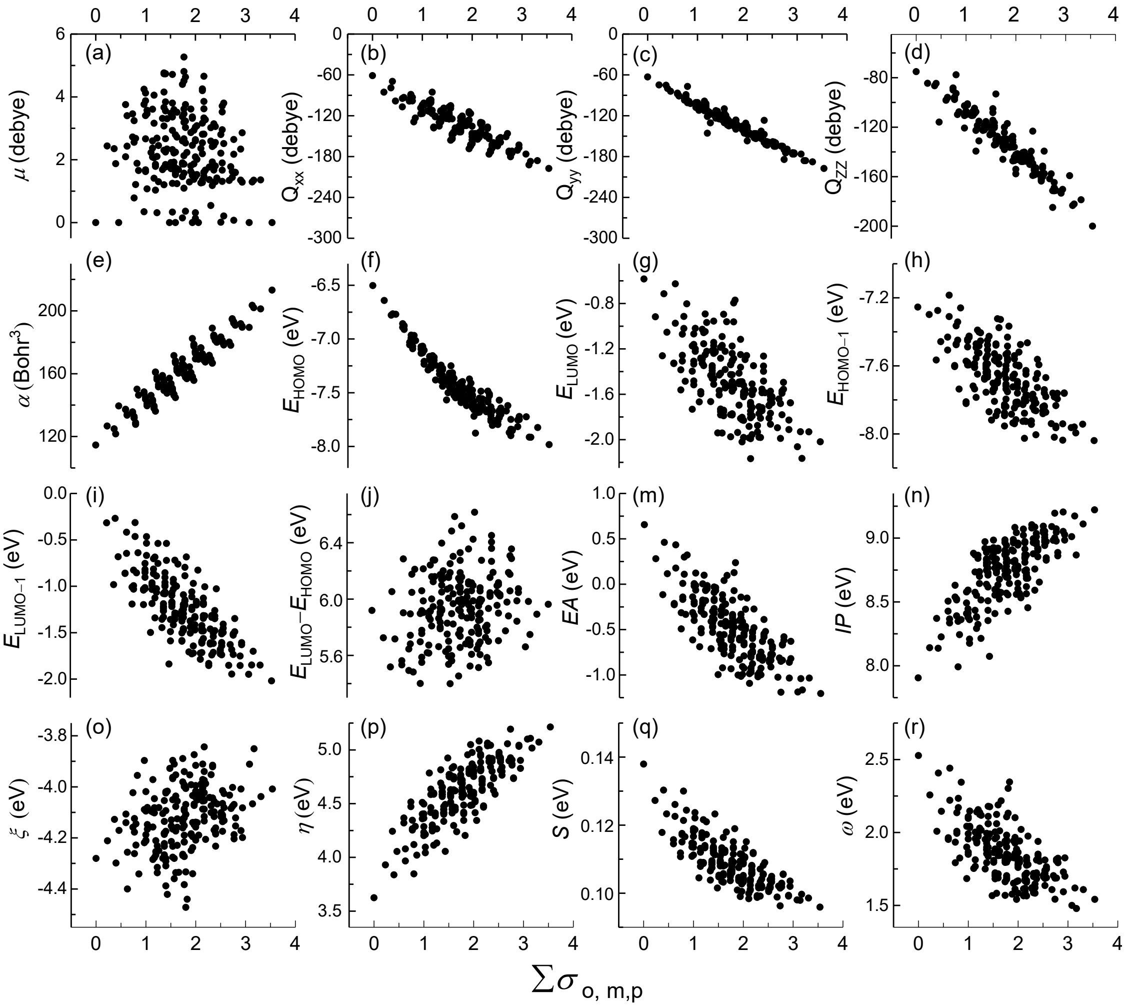

Figure S2: The relationship of 16 quantum chemical descriptors and $\sum\sigma_{o, m, p}$ for PCBs congeners.

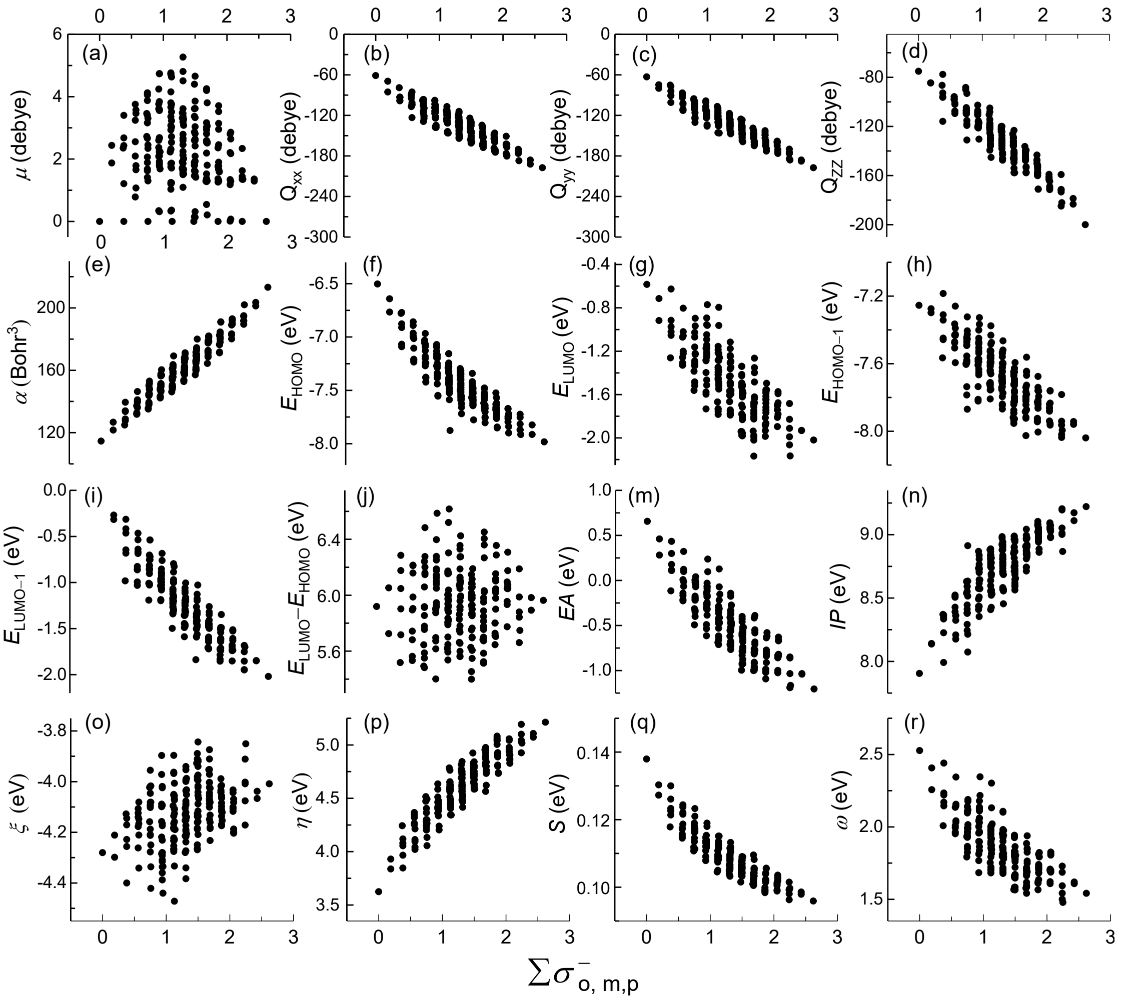

Figure S3: The relationship of 16 quantum chemical descriptors and $\sum\sigma^{-}_{o,\,m,\,p}$ for PCBs congeners.

molecules

MDPI

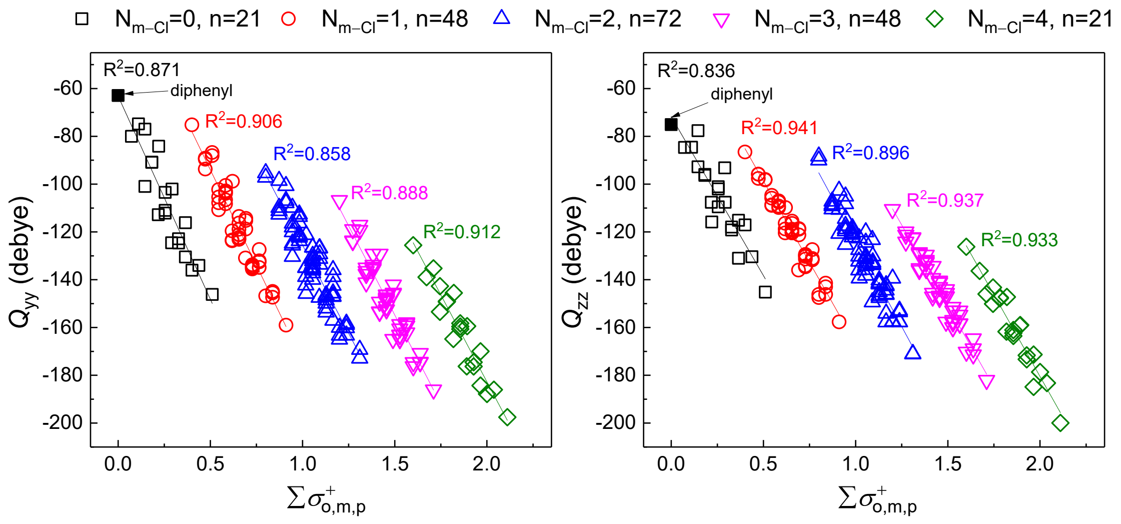

Figure S4: The relationship of $Q_{yy/zz}$ and $\sum\sigma^+_{o,\ m,\ p}$ for PCBs congeners.

molecules

MDPI

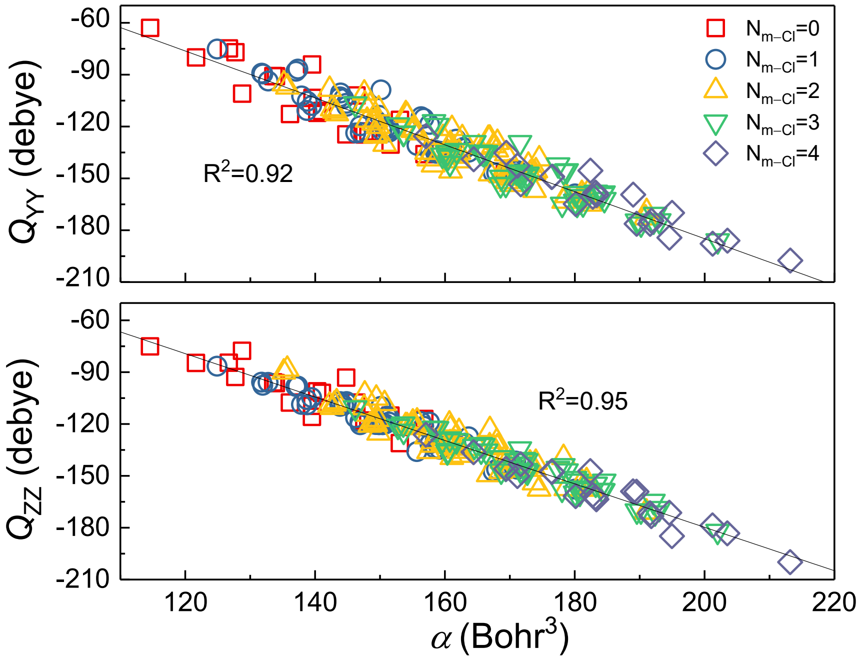

Figure S5: The relationship of α and $Q_{yy/zz}$ for PCBs congeners.

molecules MDPI

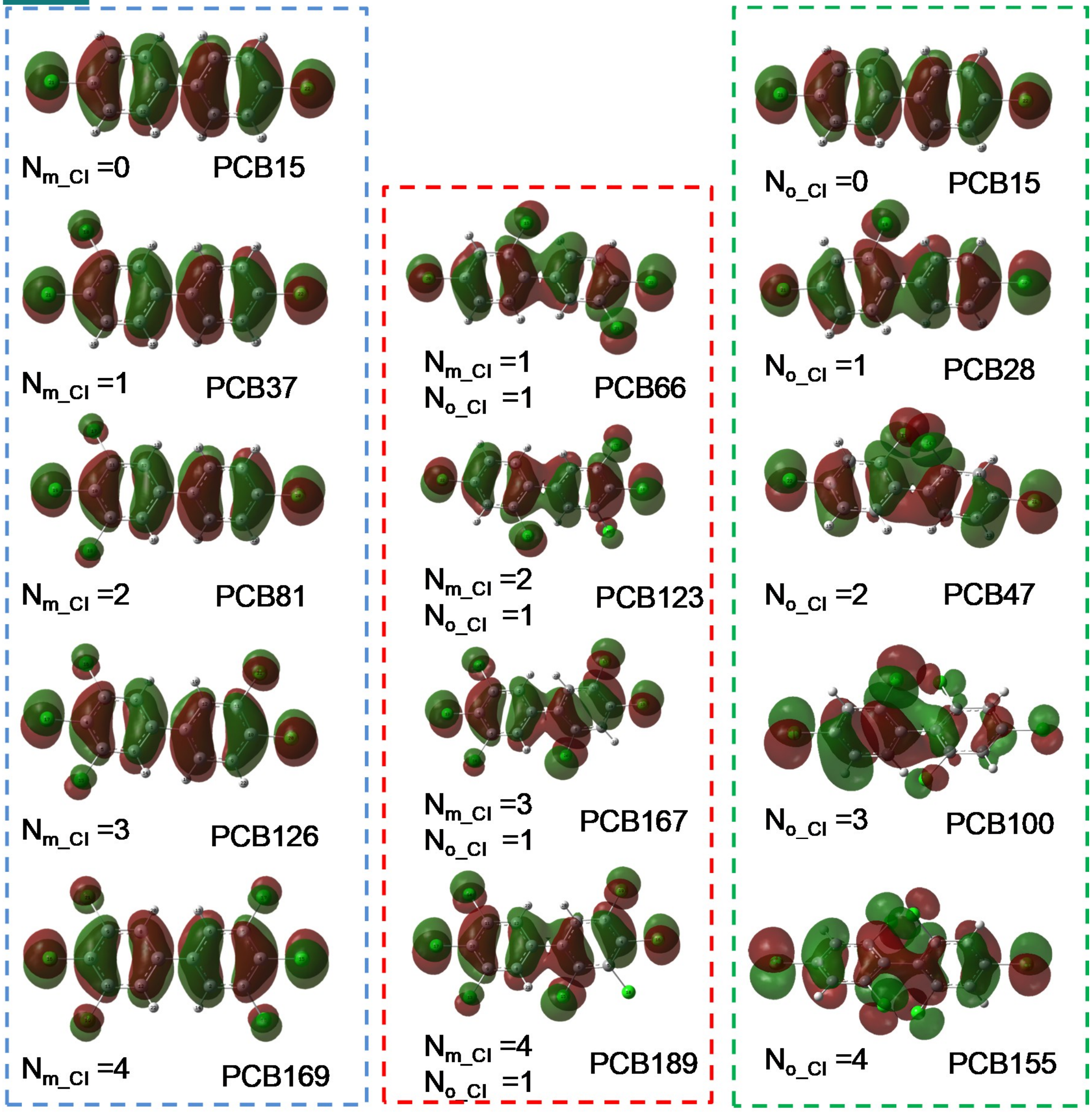

Figure S6: The HOMO distribution of a series of PCBs congeners with different N_{m_Cl} numbers at meta position (0–4) and N_{o_Cl} at ortho position (0–4) for Cl substitutes. The entire left column referred to our previous study [14].

molecules

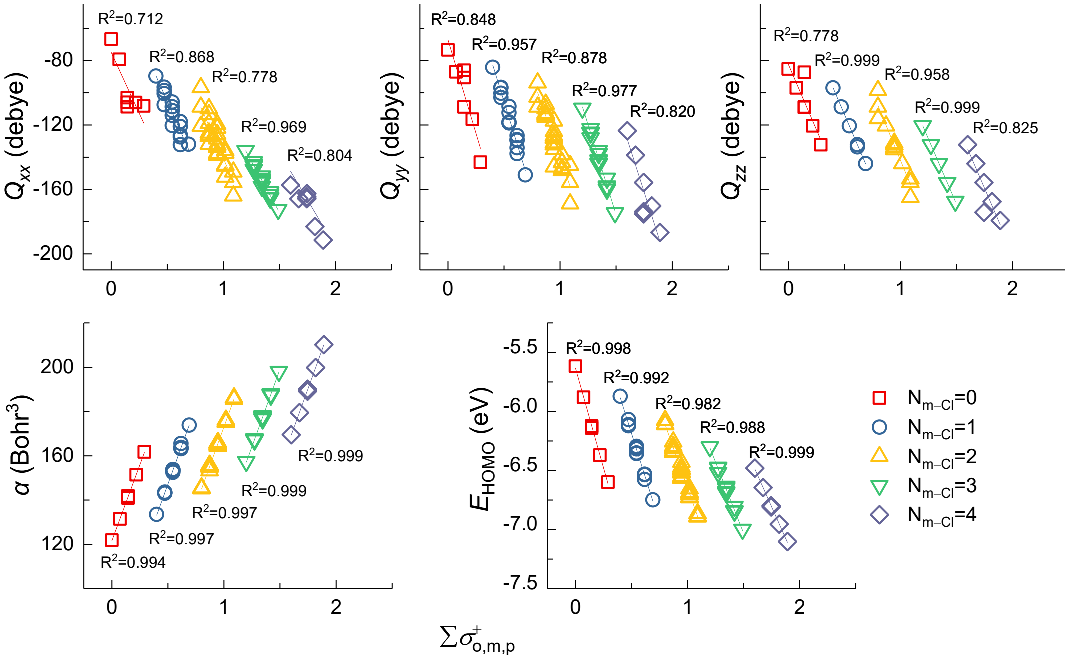

Figure S7: The relationship of $\sum\sigma^{+}_{o,\ m,\ p}$ and $Q_{xx/yy/zz}$, α and E_{HOMO} for PCDDs congeners. The $\sum\sigma^{+}_{o,\ m,\ p}$ and α referred to our previous study [14].

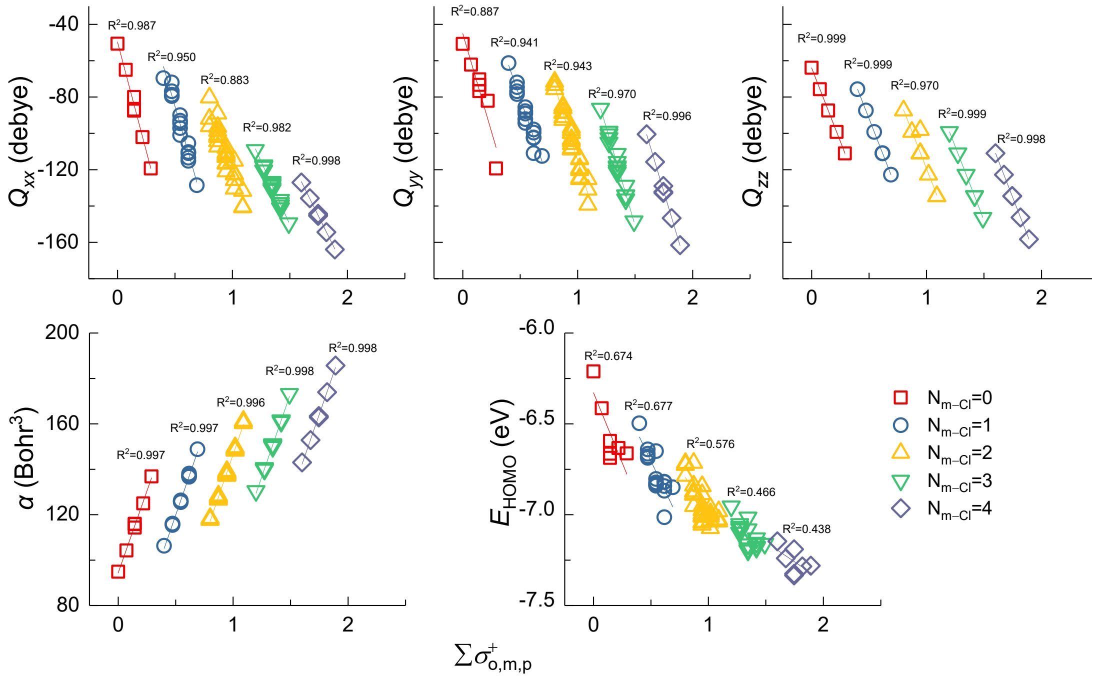

Figure S8: The relationship of $\sum\sigma^{+}_{o,\ m,\ p}$ and $Q_{xx/yy/zz}$, α and E_{HOMO} for PCNs congeners.

References

1. Hansch, C.; Leo, A.; Taft, R., A survey of Hammett substituent constants and resonance and field parameters. *Chem Rev* **1991,** 91, (2), 165-195.
2. Hammett, L. P., Some Relations between Reaction Rates and Equilibrium Constants. *Chem Rev* **1935,** 17, (1), 125-136.
3. Hansch, C.; Leo, A.; Unger, S. H.; Kim, K. H.; Nikaitani, D.; Lien, E. J., Aromatic substituent constants for structure-activity correlations. *J Med Chem* **1973,** 16, (11), 1207-1216.
4. Lee, Y.; Von Gunten, U., Quantitative structure–activity relationships (QSARs) for the transformation of organic micropollutants during oxidative water treatment. *Water Res* **2012,** 46, (19), 6177-6195.
5. Jonsson, M.; Lind, J.; Eriksen, T. E.; Merényi, G., O–H bond strengths and one-electron reduction potentials of multisubstituted phenols and phenoxyl radicals. Predictions using free energy relationships. *Journal of the Chemical Society, Perkin Transactions 2* **1993**, (9), 1567-1568.
6. Hansch, C.; Leo, A.; Hoekman, D., *Exploring QSAR: fundamentals and applications in chemistry and biology*. American Chemical Society Washington, DC: 1995; Vol. 557.
7. Anderson, P. N.; Hites, R. A., ·OH radical reactions: The major removal pathway for polychlorinated biphenyls from the atmosphere. *Environmental Science and Technology* **1996,** 30, (5), 1756-63.
8. Atkinson, R.; Aschmann, S. M.; Pitts, J. N., Kinetics of the reactions of naphthalene and biphenyl with hydroxyl radicals and with ozone at 294±1 K. *Environ Sci Technol* **1984,** 18, (2), 110-113.
9. Atkinson, R., Atmospheric chemistry of PCBs, PCDDs and PCDFs. In *Chlorinated organic micropollutants: Issues in environment science and technology*, Mester, R. E., Harrison, R.M., Ed. Royal Society of Chemistry: Cambridge, 1996; Vol. 6, pp 53-72.
10. Kwok, E. S.; Atkinson, R.; Arey, J., Rate constants for the gas-phase reactions of the ·OH radical with dichlorobiphenyls, 1-chlorodibenzo-*p*-dioxin, 1,2-dimethoxybenzene, and diphenyl ether: Estimation of ·OH radical reaction rate constants for PCBs, PCDDs, and PCDFs. *Environ Sci Technol* **1995,** 29, (6), 1591-8.
11. Huang, J.; Yu, G.; Zhang, Z.-l., Application of TLSER method in predicting the aqueous solubility and n-octanol/water partition coefficient of PCBs, PCDDs and PCDFs. *Journal of Environmental Sciences* **2004,** 16, (1), 21-29.
12. Yang, G. Y.; Yu, J.; Wang, Z. Y.; Zeng, X. L.; Ju, X. H., QSPR Study on the Aqueous Solubility (− lgSw) and n-Octanol/Water Partition Coefficients (lgKow) of Polychlorinated Dibenzo-p-dioxins (PCDDs). *QSAR Comb Sci* **2007,** 26, (3), 352-357.
13. Kim, M.; Li, L. Y.; Grace, J. R., Predictability of physicochemical properties of polychlorinated dibenzo-*p*-dioxins (PCDDs) based on single-molecular descriptor models. *Environ. Pollut.* **2016,** 213, 99-111.
14. Luo, S.; Wei, Z.; Spinney, R.; Yang, Z.; Chai, L.; Xiao, R., A novel model to predict gas–phase hydroxyl radical oxidation kinetics of polychlorinated compounds. *Chemosphere* **2017,** 172, 333-340.

Sample Availability: Samples of the compounds ...... are available from the authors.

© 2018 by the authors. Submitted for possible open access publication under the terms and conditions of the Creative Commons Attribution (CC BY) license (http://creativecommons.org/licenses/by/4.0/).
